# LCLAT1 regulates cardiolipin composition, mitochondrial phenotype, Lin28A, and oncogenic signaling networks in ETMR

**DOI:** 10.1093/noajnl/vdaf228

**Published:** 2025-10-21

**Authors:** Evangelos Liapis, Allison Maas, Kelly C O’Neill, Adele Ponzoni, Tara Lozy, Annapurna Pamreddy, Francesca M Cozzi, Brent T Harris, Derek Hanson, Claire L Carter

**Affiliations:** Center for Discovery and Innovation, Hackensack Meridian Health, Nutley, NJ; Center for Discovery and Innovation, Hackensack Meridian Health, Nutley, NJ; Center for Discovery and Innovation, Hackensack Meridian Health, Nutley, NJ; Center for Discovery and Innovation, Hackensack Meridian Health, Nutley, NJ; Center for Discovery and Innovation, Hackensack Meridian Health, Nutley, NJ; Center for Discovery and Innovation, Hackensack Meridian Health, Nutley, NJ; Center for Discovery and Innovation, Hackensack Meridian Health, Nutley, NJ; Departments of Neurology and Pathology, Georgetown University Medical Center, Washington, DC; Center for Discovery and Innovation, Hackensack Meridian Health, Nutley, NJ; Joseph M. Sanzari Cancer Center, Hackensack University Medical Center, Hackensack, NJ; Department of Pediatrics, Hackensack Meridian School of Medicine, Nutley, NJ; Center for Discovery and Innovation, Hackensack Meridian Health, Nutley, NJ; Department of Pathology, Hackensack Meridian School of Medicine, Nutley, New Jersey

**Keywords:** cardiolipins, ETMR, LCLAT1, mitochondrial dynamics and bioenergetics, pediatric brain tumors

## Abstract

**Abstract:**

BackgroundEmbryonal tumor with multilayered rosettes (ETMR) is an aggressive pediatric brain tumor that carries a poor prognosis, and there is currently no standard of care. Dysregulated mitochondrial bioenergetics and dynamics have been associated with the progression of diverse cancers. Cardiolipins are mitochondrial-specific lipids, and their fatty acid composition has been shown to regulate mitochondrial structure and function. Despite the known functional significance of cardiolipins, their structure-specific accumulation in relation to mitochondrial phenotypes in ETMR remains ill-defined.

**Methods:**

Spatial lipidomic profiles in patient samples and 3D models were determined using mass spectrometry imaging. Cell proliferation and mitochondrial bioenergetics and dynamics were characterized using immunohistochemistry, transmission electron microscopy, Western blotting, and metabolic assays. LCLAT1 KD was carried out using siRNA.

**Results:**

We detected a structure-specific accumulation of cardiolipins and increased expression of the cardiolipin acyl chain remodeling enzyme, lysocardiolipin acyltransferase 1 (LCLAT1), within proliferating tumor cells in patient samples and the 3D tumorspheres. Orthogonal imaging techniques correlated the structure-specific accumulation of cardiolipin with fragmented mitochondria displaying aberrant cristae structure, altered mitochondrial dynamics, decreased expression of respiratory chain enzymes, and a more glycolytic phenotype. LCLAT1 KD altered cardiolipin profiles, reduced growth and proliferation, decreased Sox2 and N-Myc expression, increased p53 and p21 expression, and increased LIN28A and Dcx expression. Additional therapeutic targeting of the fragmented mitochondrial phenotype identified also resulted in selective inhibition of ETMR growth and viability.

**Conclusions:**

Our findings provide novel insight into ETMR biology based on mitochondrial phenotypes and the fatty acid composition of the multifunctional mitochondrial-specific lipid, cardiolipin.

Key Points

- Our findings identified new mechanistic insights into ETMR biology.
- LCLAT1-driven cardiolipin remodeling regulates tumor suppressors, oncoproteins, and doublecortin.
- Cardiolipin acyl chain modulation offers potential as a new therapeutic approach in ETMR.

Importance of the study

By integrating spatial lipidomics with orthogonal imaging and functional assays, we identified new biological and bioenergetic features in ETMR relating to tumor growth and aggression. We found an accumulation of cardiolipins, the multifunctional mitochondrial-specific lipid, and its acyl chain remodeling enzyme LCLAT1. These alterations were determined to be intrinsic to the proliferating tumor cells in patient samples and the patient derived model. The mitochondrial phenotype we identified displayed excessive fragmentation, aberrant cristae architecture, reduced OxPhos complex expression, diminished mitochondrial respiratory capacity, and a shift to a more glycolytic phenotype. LCLAT1 knockdown altered cardiolipin composition, suppressed ETMR growth in vitro, regulated major oncogenic and tumor suppressor pathways, enhanced LIN28A and Dcx expression and partially restored mitochondrial morphology. Additionally, pharmacological targeting of the upregulated mitochondrial fission pathway selectively reduced ETMR cell viability in a dose-dependent manner. Our findings identify multiple new avenues for therapeutic intervention based on targeting the mitochondrial phenotype in this aggressive pediatric brain tumor.

Embryonal tumor with multilayered rosettes (ETMR) is an aggressive pediatric CNS tumor which carries a poor prognosis.^1–3^ ETMR most often occurs in children under the age of four, with a median age of diagnosis of 26 months.^4^ ETMR presents with low inter-tumoral genetic heterogeneity, yet with diverse histological features and developmental locations within the brain.^4^^,^^5^ Retrospective studies have demonstrated that 43% of patients diagnosed with ETMR had an event-free survival of 1–7 months, with 25–30% of patients progressing within 24 months despite aggressive therapy.^4^^,^^6^ The mechanisms driving therapeutic resistance in ETMR are unknown, and there are currently no multi-agent regimens that have demonstrated superiority.^4^ Data has demonstrated an improved 2-year event-free survival of up to 66% in patients treated with gross total resection, adjuvant high-dose chemotherapy, and radiotherapy.^4^^,^^6^^,^^7^ While this combination may improve clinical outcomes, the use of multiple aggressive chemotherapeutics and radiation causes extensive systemic toxicity. Patients can suffer from sepsis, organ failure, and succumb to the universally cytotoxic treatment.^8^^,^^9^ Those that do survive these aggressive tumors can experience significant cognitive impairment and diminished quality of life.^10–12^ New treatment approaches incorporating pre-clinical data and the known biology of ETMR are urgently needed.

Cardiolipins (CLs) are unique mitochondrial-specific phospholipids that contain four acyl chains.^13^ The composition of the different fatty acyl chains within the lipid backbone structure has been shown to regulate mitochondrial function and dynamics.^14–18^ Mechanistically, these encompass the assembly and stability of mitochondrial respiratory chain supercomplexes,^19^^,^^20^ cristae organization,^15^^,^^21^ regulation of mitophagy,^22^ and regulation of mitochondrial fusion and fission processes^23^ through direct interactions with Opa1^24^^,^^25^ and Drp1,^26–29^ respectively. Dysregulated CL profiles and corresponding alterations in mitochondrial processes contribute to the initiation and/or progression of Barth syndrome, Parkinson’s disease, metabolic diseases, age-related diseases, and cancer.^14^^,^^30–32^ Despite their known involvement in the regulation of numerous physiological and pathological mitochondrial processes, the role of CLs in pediatric brain tumor growth and aggression remains ill-defined.^33–35^

In this study, we used mass spectrometry imaging (MSI) in combination with orthogonal techniques to study CL accumulation in relation to mitochondrial phenotypes in ETMR patient samples and the patient-derived cell line BT183. We used a neural stem cell (NSC) model as a control, given its success in identifying CNS toxic compounds during drug screening experiments, and its potential to aid in the identification of tumor-specific stem cell alterations.^36^ We show that structure-specific CLs accumulated in actively proliferating embryonal tumor cells that contained fragmented mitochondria in both patient samples and the BT183 cell model. These alterations were further shown to correlate with reduced expression of respiratory chain complexes, aberrant cristae formation, and a more glycolytic phenotype when compared with NSCs. We identified lysocardiolipin acyltransferase 1 (LCLAT1, also known as ALCAT1, LYCAT, or AGPAT8), the CL acyl chain remodeling enzyme, as a potential new therapeutic target in ETMR, and we demonstrate efficacy in targeting LCLAT1-driven CL remodeling and the mitochondrial phenotype detected. These findings provide important phenotypic information for ETMR and offer new avenues for therapeutic investigation.

## Methods

### Patient Samples

Five frozen ETMR patient samples were provided by the Children’s Brain Tumor Network (CBTN).^37^ All samples were de-identified and obtained with informed consent from the patients’ legal guardians. Sample collection was approved by the Institutional Review Board (IRB) at CBTN affiliated hospitals. Formalin-fixed paraffin embedded (FFPE) ETMR tumor specimens were obtained from the Joseph M. Sanzari Children’s Hospital at the Hackensack University Medical Center through IRB protocol number Pro2023-0087. Patient information is listed in Supplementary Table S1. Sectioning of ETMR patient samples is described in the Supplementary Materials and Methods.

### Cell Lines and Cell Culture

BT183 and NSC cells were grown in ultra-low attachment (ULA) flasks in Complete NeuroCult medium, supplemented with EGF, bFGF, and heparin, as described in Supplementary Materials and Methods.

### Ki67 Labeling Index Scoring

Quantification of the Ki67^+^ labelling index (LI) in ETMR and in BT183 and NSC cryosections was carried out using automated cell counting in ImageJ, as described in Supplementary Materials and Methods.

### Preparation of BT183 and NSC Spheroids for Transmission Electron Microscopy (TEM)

BT183 and NSC tumor/neurospheres were fixed in 2% paraformaldehyde and 2.5% gluteraldehyde in 0.1 M Cacodylate buffer, as described in the Supplementary Materials and Methods.

### BT183 and NSC Spheroid Growth Kinetics

Growth kinetics of BT183 and NSC spheroids were determined in ImageJ using phase contrast images acquired with Celigo Imaging Cytometer, as described in Supplementary Materials and Methods.

### Mass Spectrometry

MALDI MSI was carried out on frozen ETMR patient samples and the 3D models using a Bruker Solarix XR 7T FTICR mass spectrometer, according to previously published protocols.^35^^,^^38^ Patient samples were annotated by a board-certified pathologist, and each H&E image was then imported into the SCiLS Lab multi-vendor software, ver. 2023c Pro (Bruker Daltonics) for co-registration with their corresponding MSI data.

CL profiles were acquired from lipid extracts of LCLAT1 siRNA knockdown and negative control BT183 cells using a Bruker Solarix XR 7T FTICR mass spectrometer. Mass spectra were acquired in the negative ion mode, averaging 50 scans per acquisition. The ion transfer optics were optimized for the CL mass range.

### Immunohistochemical Staining

A list of materials used for IHC staining of the FFPE sections and cryosections is provided in Supplementary Table S2. Sample preparation for IHC staining was carried out according to standard protocols for FFPE and frozen sections. Primary and secondary antibodies and their concentrations are listed in Supplementary Table S3.

### Microscopy and Image Analysis

IHC-stained frozen and FFPE ETMR sections and BT183/NSC spheroid cryosections as well as MitoView™ Fix 640-stained BT183 and NSC monolayers were imaged and analyzed as described in Supplementary Materials and Methods.

### Western Blotting

Cell lysate preparation, denaturing SDS-PAGE electrophoresis, protein transfer, total protein staining, blocking, primary and secondary antibody incubation, and chemiluminescence detection are described in detail in Supplementary Materials and Methods. Supplementary Table S4 lists antibodies and detailed experimental parameters.

### Analysis of RNA Expression Profiles

*RNA expression profiles of genes of interest were analyzed from ETMR and 6 additional pediatric brain tumors in comparison to normal brain regions using the Open Pediatric Cancer (OpenPedCan) project (v15)* ^39^  *queried through the* PedcBioPortal (https://pedcbioportal.org). OpenPedCan is an extension of the Open Pediatric Brain Tumor Atlas (OpenPBTA)^40^ and contains processed multi-omic data from ∼24,002 tumors and 23,893 normal biospecimens. These datasets have been collected from multiple sources including seven cohorts of pediatric tumors, The Cancer Genome Atlas (TCGA), and the Genotype-Tissue Expression (GTeX) project.

### Measurement of OCR and ECAR in BT183 and NSC Spheroids

The Oxygen Consumption Rate (OCR) and Extracellular Acidification Rate (ECAR) were determined using the MitoXpress^®^ Xtra (MitoX) oxygen consumption and the pH-Xtra™ (pHX) glycolysis assay kits (Agilent), respectively, following the manufacturer’s recommendations, as described in Supplementary Materials and Methods.

### LCLAT1 siRNA Knockdown

BT183 cells were seeded at a density of 600,000 cells per well in a 6-well plate and transfected with either the Silencer™ Select Negative Control No. 1 siRNA (ThermoFisher) or LCLAT1 Silencer^®^ Select (Human) siRNA (ThermoFisher) at a final siRNA concentration of 20 nM, using GenMute siRNA Transfection Reagent (SignaGen Laboratories) per the manufacturer’s instructions. Several independent transfection experiments were performed using the following transfection schedules; *Schedule 1*: Cells were transfected twice on days 0 and 2 and harvested for Western blot analysis on day 3. *Schedule 2*: Cells were transfected on days 0, 2, and 4 and harvested for Western blot analysis on day 7. *Schedule 3*: Three rounds of transfection were performed on days 0, 3, and 6, and cells were harvested on day 7 for spheroid growth analysis and on day 10 for mass spectrometry lipid profiling, Western blot analysis, and quantitative assessment of cell confluence, total cell number, and proliferative activity (Ki67+ LI), as outlined in Supplementary Methods.

### Mdivi-1 Treatment of BT183 and NSC Spheroids

BT183 and NSCs were seeded in round bottom 96-well ULA plates (Corning) at a density of 2,500 and 5,000 cells per well, respectively. Spheroids were treated with vehicle (DMSO) or Mdivi-1 (Sigma, #M0199) at a dose range of 5–200 μM when they reached a diameter of approx. 500 µm. Cell viability was measured after 72 h of treatment with Mdivi-1 using the Alamar Blue assay, as detailed in Supplementary Materials and Methods.

### Data and Statistical Analyses

MSI data was processed using the unsupervised multivariate analysis, probabilistic latent semantic analysis (pLSA), and principal component analysis. Ions in the pLSA score images were identified using accurate mass-based lipid assignments in HMBD and LipidMaps databases. Further identification for select lipids was carried out following MS fragmentation studies. The discriminating capability for CLs in ETMR was further determined using ROC-AUC, and statistical significance was determined using *t*-test.

Statistical differences in relative protein expression, mitochondrial morphological characteristics, and OCR/ECAR between BT183 and NSCs were assessed by Welch’s *t*-test in GraphPad Prism (GraphPad Software, San Diego, CA).

## Results

### Patient Sample Pathology, Proliferation Index, and Multivariate Analysis of MS Imaging Data

**Figure 1.**
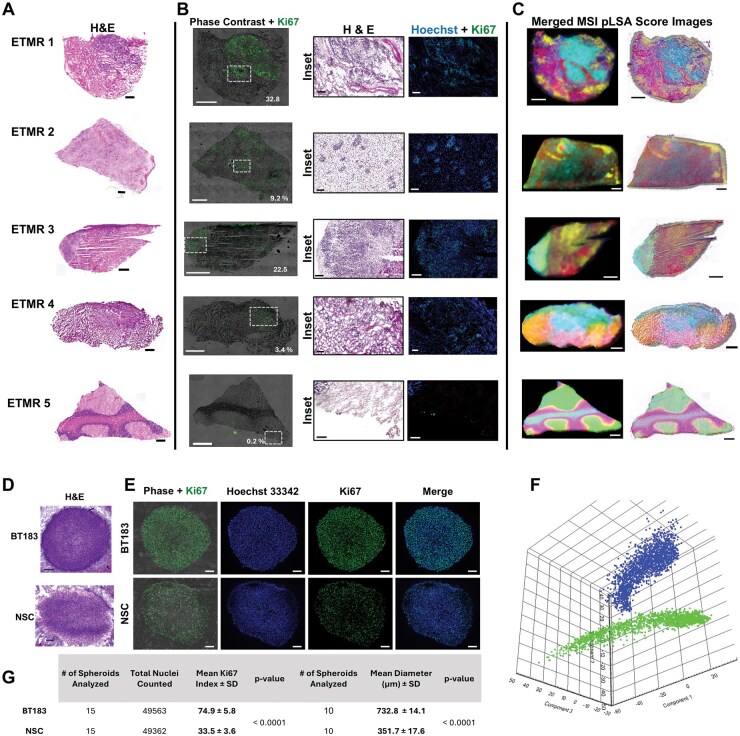
(A) H&E-stained sections of patient samples. (B) Ki67 immunofluorescent staining with corresponding phase contrast image. (C) Mass spectrometry imaging data presented as merged pLSA score images on their own and superimposed with their corresponding H&E-stained sections. Scale bars: whole tumor sections = 1 mm, insets = 50 µm. (D) H&E-stained sections of BT183 and NSC 3D spheroids. Scale bars = 100 µm. (E) IHC staining of BT183 and NSC 3D spheroid cryosections against Ki67. Left panels show merged phase contrast and Ki67 immunofluorescence (green). Hoechst 33342 was used as a nuclear counterstain. Scale bars = 100 µm. (F) Principal component analysis of MSI data obtained from BT183 (blue) and NSC (Green). (G) Ki67 labeling indices and mean spheroid diameter for BT183 and NSC spheroids at day 7 post-seeding (*n* = 15 spheroids per cell type).

The five frozen ETMR patient samples provided by the Children’s Brain Tumor Network encompassed 1 progressive tumor (tumor progressed during treatment), 3 primary tumors, and 1 recurrent tumor. We carried out H&E staining and mass spectrometry imaging on the same sections and Ki67 immunostaining on serial sections, to correlate lipidomic accumulation with tumor microenvironment histopathology, and tumor cell proliferation. The H&E-stained sections demonstrated intra- and intertumoral heterogeneity with varying Ki67 immunoreactivity (Figure 1A and B). Higher magnification images with more detailed histological description for each sample are shown in Supplementary Figures S1–S5.

MSI data was initially processed using the unsupervised multivariate analysis tool, pLSA. This automatically reduced the thousands of mass spectra collected across each tissue section and processed them into score images based on the intensity and spatial distribution of each ion detected. The individual score images were then processed using a false color scheme and presented as merged images alone and superimposed with their corresponding H&E-stained sections (Figure 1C). The MSI score images segmented with the histopathological presentation and cellular proliferation for each sample (Figure 1A–C). An example of this is demonstrated more clearly in Supplementary Figure S6A–E. Data from the Ki67 and H&E staining in correlation with the merged pLSA score image and example mass spectra from the predominant score images (color-coded), correlating to high and low tumor cell regions, show that cardiolipin and glycolipid profiles differ between the two cellular regions. Spectral loadings from the pLSA score images in high vs. low tumor regions were further processed using ROC-AUC discriminative analysis and further confirmed the pathology- and structure-specific accumulation of cardiolipins (CLs) within the actively proliferating tumor cell populations. We next set out to determine whether the CLs that accumulated in the actively proliferating tumor cells in patient samples were also upregulated in the patient-derived primary ETMR cell line, as described below.

### ETMR and NSC Spheroid Pathology, Growth Kinetics, and Multivariate Analysis of MS Imaging Data

The primary ETMR cell line, designated BT183, was originally derived from a treatment naive 2-year-old male patient and has been shown to maintain key genetic and histologic features, including the C19MC amplification and LIN28A overexpression that are required for diagnosis.^1^^,^^3^ The BT183 cell line is well characterized and grows readily *in vitro* as 3D tumorspheres and *in vivo* following orthotopic transplantation.^3^ BT183 has previously been utilized in preclinical studies that identified more effective chemotherapeutic regimens, which have been translated to clinic.^8^^,^^41^ We aimed to determine whether CL, the functional mitochondrial-specific lipids that were accumulated in the tumor cells in patient samples, were also present in the cell model, thereby validating the model for CL-based functional studies. We additionally carried out a comparison of BT183 and NSCs, with an aim to identify tumor-specific stem cell alterations that could hold potential as targeted therapeutic options. Immunofluorescent characterization of the 3D models showed that both had strong immunoreactivity against the neural stem cell markers Sox2, Nestin, and Pax6 (Supplementary Figure S7), in agreement with previously published data.^3^^,^^42^ BT183 also demonstrated strong immunostaining for the ETMR diagnostic pluripotency marker LIN28A (Supplementary Figure S7). Representative H&E-stained sections and Ki67 immunostaining of BT183 and NSC spheroids are shown in Figure 1D and E, respectively. MSI and principal component analysis of the resulting data demonstrated that the mass spectral profiles are clearly separated into two clusters for BT183 and NSCs (Figure 1F). Following ROC-AUC comparative analysis to identify discriminative lipids, these data were correlated with the MSI data from the patient samples. The embryonal tumor cell populations in the BT183 spheroid demonstrated ∼two-fold increase in Ki67 LI when compared with the NSCs (Figure 1G). Ki67 is reported to be a graded rather than a binary marker of cell proliferation due to its accumulation and degradation during different stages of the cell cycle.^43^ The two-fold increase in Ki67 LI in BT183 compared to NSCs is attributed to faster replication rates in the embryonal tumor cells, which is further supported by the 3D growth data presented in Figure 1G. BT183 displayed significantly larger spheroid diameters at day 7 post-seeding.

### CL Composition is Altered in ETMR

**Figure 2.**
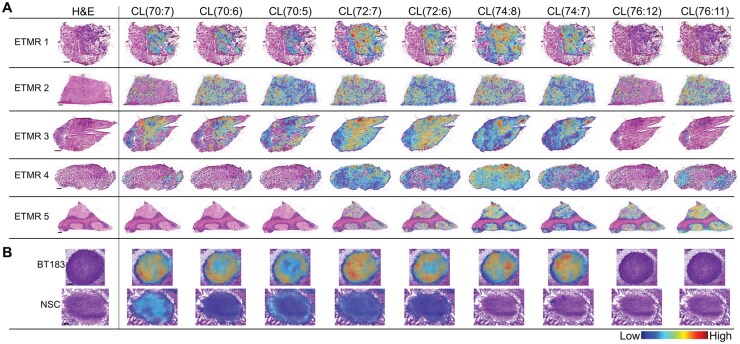
Structure-specific cardiolipin accumulation in ETMR. Example MS images of cardiolipin (CL) lipid species in (A) patient samples and (B) the BT183/NSC spheroid models. CLs that discriminated the high vs. low tumor cell regions and BT183 vs. NSCs were identified with a ROC-AUC between 0.73 and 0.95 and *P*-values <.001–.003, *t*-test.

In the patient samples, structure-specific CLs were shown to accumulate within the medium and high-density tumor cell areas that were Ki67^+^. Their accumulation within the embryonal tumor cells in patient samples correlated with their increased accumulation in BT183 when compared with NSCs (Figure 2A and B). CLs with a fatty acid composition of higher carbon numbers and double bonds were not detected within the tumor cells or the normal stem cell model but were detected within the molecular layer of the normal-appearing cerebellum in ETMR 5. The CL alterations detected represent intrinsic changes in the tumor stem cell populations, as they occur in the patient samples and the patient-derived cell model, despite the differences in growth conditions and microenvironmental cues (*in vitro* vs. *in vivo*). This finding validates the model for studying the functional role of CL composition in ETMR biology.

### The CL Fatty Acid Remodeling Enzyme LCLAT1 is Overexpressed in ETMR

**Figure 3.**
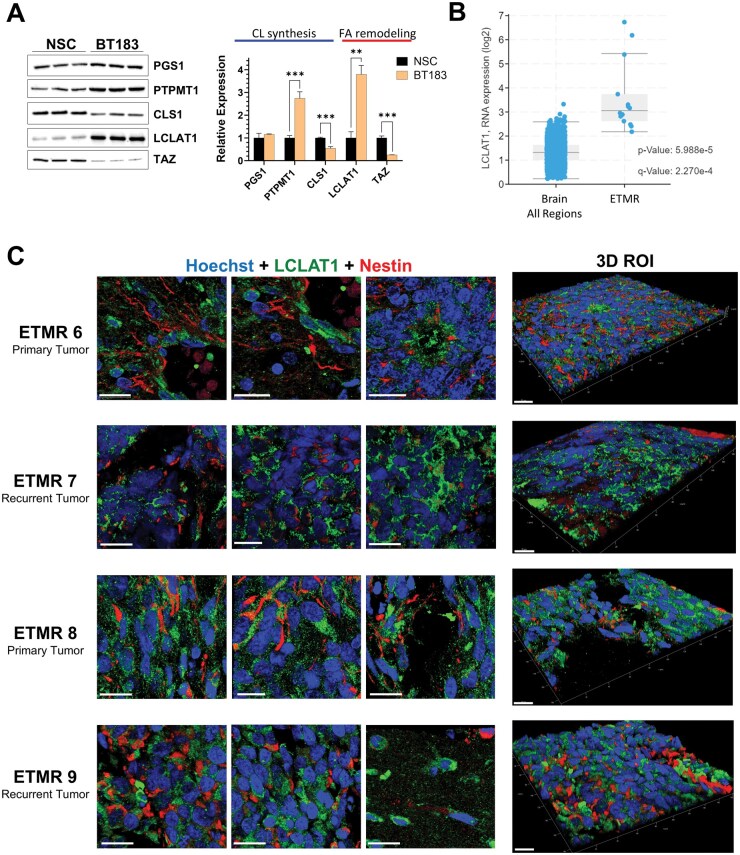
(A) Expression of CL synthesizing and remodeling enzymes in BT183 compared to NSCs grown in 3D. Data represents the mean fold change in protein expression in BT183 relative to NSCs ± SD (*n* = 3–4 protein bands per cell type). *P < .05, **P < .01, ***P < .001, Welch’s *t*-test. Data were normalized to total protein (Suppl. Figure S8). (B) LCLAT1 RNA expression data exported in normal brain vs. ETMR. **C)** Immunofluorescence staining of LCALT1 (green) and Nestin (red). Hoechst 33342 was used as a nuclear marker. Scale bars = 20 µm.

As the structure-specific accumulation of cardiolipins is determined by the (in)action of several synthesizing and remodeling enzymes, we next sought to correlate CL alterations with the relative expression of these enzymes. We first focused on determining their expression levels in BT183 compared to NSCs, using western blotting (Figure 3A;  Supplementary Figure S8 for total protein images). For the enzymes involved in the multistep process of cardiolipin synthesis, phosphatidylglycerol phosphate synthase (PGS1) expression was detected at similar levels. The expression levels of protein tyrosine phosphatase mitochondrial 1 (PTPMT1), the enzyme involved in the penultimate step of CL biosynthesis, was significantly elevated in BT183 compared to NSCs. CL synthase (CLS1), the enzyme involved in the final step of nascent CL synthesis, showed significantly lower expression in the BT183 spheroids compared to the NSCs. Among the CL acyl chain remodeling enzymes investigated, the expression of lysocardiolipin acyltransferase-1 (LCLAT1) was significantly higher in BT183 compared to NSCs. In contrast to LCLAT1, expression levels of the transacylase, tafazzin (TAZ), were markedly lower. Overexpression of LCLAT1 and subsequent CL fatty acyl chain remodeling is a known pathogenic driver in a number of metabolic diseases and non-CNS adult cancers.^32^^,^^44^ To determine whether the increased expression of LCLAT1 observed *in vitro* was also present in ETMR patient samples, we first queried RNA expression data in the OpenPedCan Project (v15) using the PedcBioPortal (https://pedcbioportal.org). We compared LCLAT1 expression in normal brain tissue (GTeX), which encompassed 2642 samples spanning 382 patients, to 17 ETMR samples from 15 patients. The results showed a significant increase in LCLAT1 expression in ETMR when compared with normal adult brain (Figure 3B). We then compared LCLAT1 expression in normal brain and a further 6 pediatric brain tumors encompassing the embryonal tumors, atypical teratoid/rhabdoid tumor, medulloblastoma, and the aggressive or hard to treat tumors, diffuse intrinsic pontine glioma, diffuse midline glioma, high grade glioma, and ependymoma. Overlapping datasets from the glioma groups were excluded. Our analysis showed an increase in LCLAT1 expression in all tumors compared to normal brain tissue (Supplementary Figure S9). It should be noted that the GTeX data is derived from adults with an age range of 21–70 years, while the age at diagnosis for the tumor groups range from <5 to 25 years, and this could be contributing to the differences in expression. As there can be discrepancies in gene expression and corresponding protein expression, we next sought to determine LCLAT1 protein expression in ETMR patient samples using IHC. We obtained FFPE blocks from 4 additional ETMR patients for IHC analysis. The resected tumors encompassed two primary and two recurrent ETMRs. The Ki67 indexing in all samples was high, 60–90%, based on the pathology report. High expression levels of LCLAT1 were detected in the embryonal tumor cells in all ETMR FFPE sections, regardless of the pathological presentation (Figure 3C;  Supplementary Figure S10). Collectively, these results demonstrate that overexpression of LCLAT1 is contributing to the acyl chain composition of the CLs detected in both the patient samples and the *in vitro* patient-derived 3D spheroid model.

### ETMR Spheroids Have Reduced Expression of OxPhos Subunits and a More Glycolytic Phenotype

**Figure 4.**
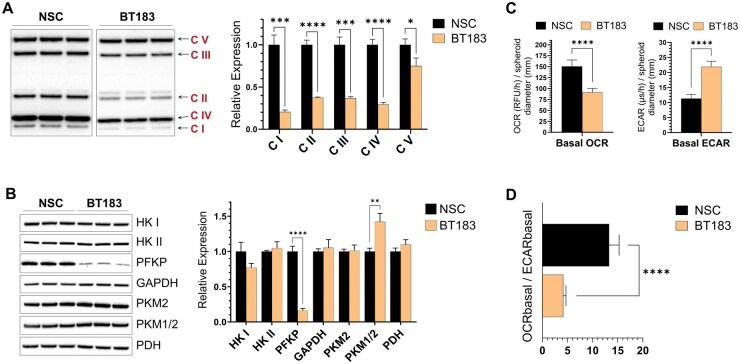
Comparative assessment of OxPhos and Glycolysis in BT183 and NSC spheroids. (A) Relative expression of ETC Complex subunits; Complex I (NDUFB8, 18 kD), II (SDHB, 29 kD), III (UQCRC2, 48 kD), IV (COX II, 22 kD), and V (ATP5A, 54 kD) in BT183 and NSC neurosphere lysates. (B) Western blot analysis of key enzymes of the glycolysis pathway in BT183 vs. NSC spheroids. Data shown in panels A-B represent the mean fold change in protein expression in BT183 relative to NSCs ± SD (*n* = 3–4 protein bands per cell type). (C) Basal Oxygen consumption rate (OCR) and Extracellular acidification rate (ECAR) for BT183 and NSC spheroids. Values for basal OCRs are expressed as MitoX probe signal intensity (RFU/h) normalized to spheroid diameter (mm) ± SD (*n* = 6), and for basal ECARs as pHX probe lifetime (µs/h) normalized to spheroid diameter (mm) ± SD (*n* = 6). (D) Ratio of basal OCR to basal ECAR in BT183 and NSC spheroids ± SD (*n* = 6). *P < .05, **P < .01, ***P < .001, ****P < .0001, Welch’s *t*-test.

The interaction of cardiolipins with mitochondrial respiratory complexes is essential for structural stability, supercomplex formation, and enzymatic activity.^19^^,^^20^ Alterations in CL composition leads to decoupling of the complexes, destabilization of the electron transport chain (ETC), and diminished OxPhos capacity.^19^^,^^20^^,^^33^ To evaluate the impact of the altered CL composition detected in ETMR on the expression of respiratory complex subunits, we performed comparative western blot analysis of BT183 and NSC spheroids. The analysis of respiratory complexes I-IV of the ETC revealed significantly lower expression in BT183 compared to NSCs (Figure 4A). Expression of complex V (ATP-synthase) was also found to be significantly lower in BT183 (Figure 4A). As cancer cells are known to undergo aerobic glycolysis, we additionally investigated the expression levels of several proteins involved in glycolysis. Among the glycolytic enzymes evaluated, we found similar expression levels of Hexokinases I and II, GAPDH, and PDH between BT183 and NSCs (Figure 4B). Total pyruvate kinase (PKM1/2) expression was significantly increased in BT183, whereas PKM2 expression was comparable between the two cell types. PFKP expression was several-fold higher in NSCs (Figure 4B).

To correlate enzyme expression with function, we next determined OxPhos and glycolytic activities in BT183 and NCS spheroids. Analysis of OCR and ECAR rates revealed significantly reduced basal OCR and higher basal ECAR in BT183 (Figure 4C). Accordingly, the ratio of basal OCR to basal ECAR was markedly lower in BT183 (Figure 4D). Combined, the findings from the protein expression and functional metabolic assays demonstrated that ETMR cells maintain a more glycolytic metabolic phenotype, coupled with diminished expression of complexes I-V, and increased expression of PKM1. Importantly, this bioenergetic phenotype was associated with the higher proliferation and growth rate in the embryonal tumor cells (Figure 2G).

### Structure-Specific CLs Accumulate in Embryonal Tumor Cells with Altered Mitochondrial Morphology and Dynamics

**Figure 5.**
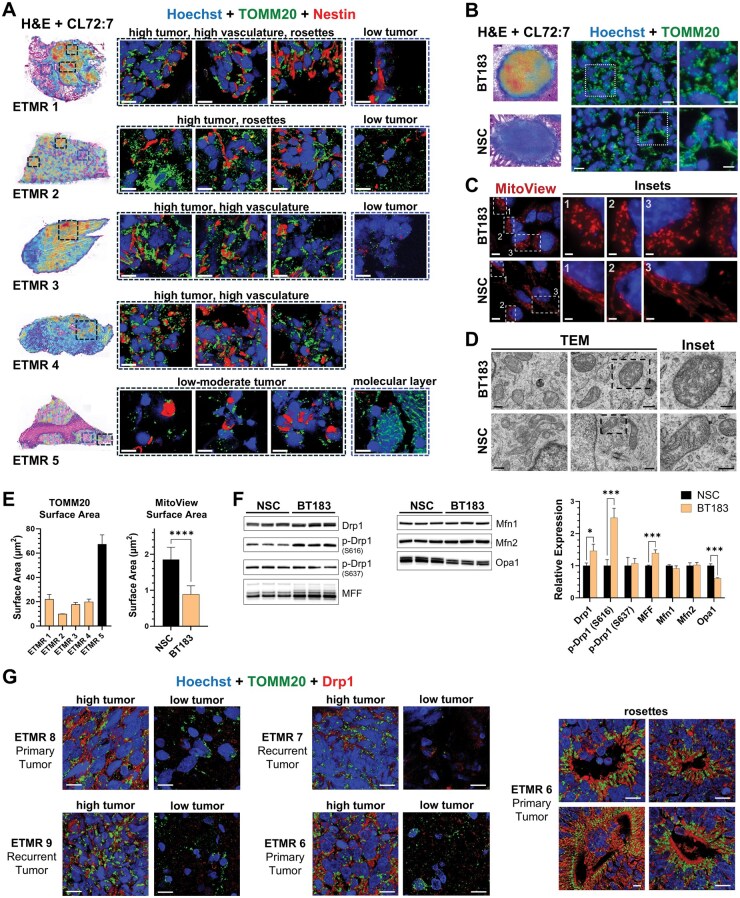
ETMR cells display fragmented mitochondrial morphology and altered expression of fission–fusion proteins. (A) Left panels: MSI and H&E merged images of whole ETMR sections showing regions of high accumulation of shorter acyl chain CL species. Right panels: Confocal images (63x) of ETMR sections immunohistochemically stained against the mitochondrial translocase TOMM20 (green) and Nestin (red), showing mitochondrial morphology and distribution in representative regions designated by black and blue dotted rectangles in the corresponding merged MSI and H&E images (left panels). Scale bars = 10 µm. (B) Left panels: Merged MSI and H&E images of representative BT183 and NSC neurospheres showing preferential accumulation of shorter acyl chain CLs in BT183. Right panels: Fluorescent micrographs showing TOMM20-stained mitochondria in BT183 and NSC. Scale bars = 10 µm. Insets: scale bars = 2.5 µm. (C) Representative images of mitochondria fluorescently stained with MitoView Fix 640 in BT183 and NSC cells grown as 2D monolayers. Scale bars = 10 µm. Insets: scale bars = 2.5 µm. Hoechst 33342 was used to visualize the nuclei in panels A-C. (D) TEM images of mitochondria in BT183 and NSC spheroids. Scale bars = 500 nm. Insets: scale bars = 250 nm. (E) 3D quantification of mean mitochondrial surface area in ETMR samples 1–5 (left panel) and 2D quantification of mean mitochondrial surface area in NSC and BT183 monolayers (right panel). (F) Comparative Western blot analysis of proteins involved in mitochondrial fission–fusion in BT183 and NSC spheroids. Bar charts depict relative protein expression normalized to total protein content. Data represents the mean fold change in protein expression in BT183 relative to NSCs ± SD (*n* = 3–4 protein bands per cell type). *P < .05, **P < .01, ***P < .001, ****P < .0001. (G) ETMR sections immunohistochemically stained against TOMM20 (green) and Drp1 (red), showing distribution in representative regions. Scale bars = 10 µm. Hoechst 33342 was used to visualize the nuclei in all fluorescent images.

CL directly interacts with both Drp1 and Opa1, and its structure-specific association with these proteins is essential for mitochondrial fission and fusion, respectively.^23^^,^^25^^,^^28^^,^^29^ Additionally, mitochondrial dynamics are intricately linked to the metabolic requirements of the cell. Thus, we sought to correlate CL composition in ETMR with mitochondrial morphology in patient samples and the 3D models (Figure 5A–F). IHC staining against the mitochondrial translocase, TOMM20, was carried out in sections within 50 µm to those used for the MSI analysis of CLs and Ki67 staining in patient samples. Mitochondria in regions of highly proliferating tumor cells with structure-specific CLs were mostly spherical structures with little elongation and interconnectivity (Figure 5A). In contrast, mitochondria in regions of low tumor cell density that contained low-to-no detection of these CLs were much fewer in number. Within the molecular layer of the cerebellum of ETMR 5, which contained longer acyl chain CLs (Figure 2), mitochondria displayed distinct elongated morphology and interconnected networks (Figure 5A, bottom panel). Mitochondria in the highly proliferating embryonal tumor cells in the BT183 spheroids also appeared as round, donut-shaped structures with no apparent elongation or connectivity. Conversely, NSC spheroids contained mainly elongated mitochondria that formed intricate intra- and intercellular networks (Figure 5B; Supplementary Figure S11A). These results agree with previously published data for synaptic and nonsynaptic mitochondria in neuronal and NSCs, which displayed predominantly elongated and interconnected mitochondrial networks.^45^^,^^46^ To enable more accurate morphometric analysis of mitochondria from single cells, 2D monolayers of BT183 and NSCs were stained with MitoView or against citrate synthase (Figure 5C;  Supplementary Figure S11B–C), which further confirmed the fragmented mitochondrial phenotype in ETMR. At the ultrastructural level, TEM analysis revealed primarily round-shaped mitochondria with aberrant cristae structure in the embryonal tumor cells of BT183, as opposed to elongated mitochondria with normal cristae morphology in the NSCs (Figure 5D;  Supplementary Figure S11D).

We then performed quantitative morphometric analysis of mitochondrial size and shape in the patient samples and 3D models (Figure 5E). Reduced mitochondrial surface area and volume are shown in high tumor cell density areas of ETMR samples 1–4. In contrast, the surface area and volume of mitochondria in the molecular layer of ETMR 5 were considerably larger (Figure 5E).

We next investigated whether the observed fragmented mitochondrial morphology in the embryonal tumor cells reflected differences in proteins that regulate mitochondrial dynamics (Figure 5F). The mean expression levels of the pro-fission enzymes Drp1, its activated form (pDrp1-S616), and MFF were significantly higher in BT183 compared to NSCs. Conversely, expression levels of the pro-fusion mediator Opa1, were significantly lower in BT183 than in NSCs. The mitofusins, Mfn1/Mfn2, were comparable between the two cell types. These findings demonstrate that both excess mitochondrial fission and reduced mitochondrial fusion contribute to the fragmented mitochondrial morphology observed in the embryonal tumor cells. We next carried out IHC staining against Drp1 in the FFPE patient samples to determine whether the increase in BT183 relative to NSCs also correlated with increased Drp1 expression in ETMR tumor cells relative to low tumor cell brain regions (Figure 5G). Image analysis revealed high Drp1 expression with partial mitochondrial co-localization in high- and moderate-density tumor regions, and in multilayered rosettes, while expression was markedly reduced in low-density tumor areas.

### LCLAT1 Knockdown in BT183 Cells

**Figure 6.**
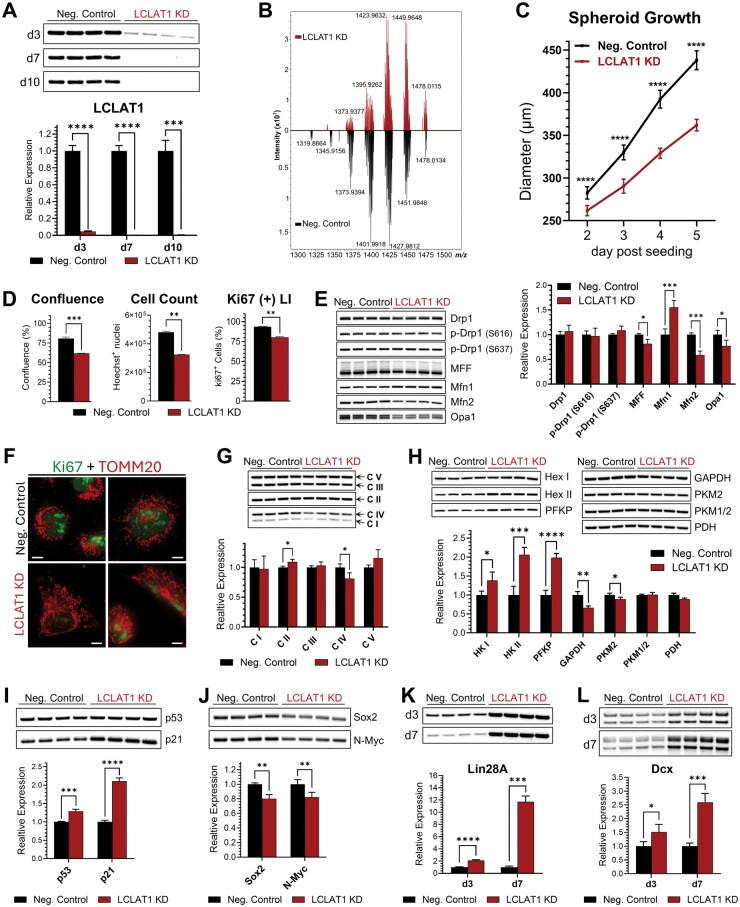
LCLAT1 knockdown in BT183 cells. (A) Western blot quantification of LCLAT1 relative expression in BT183 cells transfected multiple times with Negative Control or LCLAT1-targeting siRNA and harvested for analysis on d3, d7, or d10 post initial transfection, as outlined in Methods section. (B) Mass spectra of cardiolipin profiles from LCLAT1 siRNA knockdown (top) and negative control (bottom) BT183 cells lipid extracts. (C) Growth kinetics for control and LCLAT1 knockdown BT183 tumorspheres over a period of 5 days. Data represent mean spheroid diameter (*n* = 10) ± SD. (D) Cell confluence (*n* = 2 plates per sample ± SEM), total cell number (*n* = 3 wells per sample ± SD), and Ki67+ LI (*n* = 3 wells per sample ± SD) estimates for control and LCLAT1 knockdown BT183 monolayers (d10), determined automatically by whole-plate imaging at 4x magnification, as described in Methods section. (E) Western blot analysis of mitochondrial fission–fusion proteins. (F) Immunocytochemical staining of control and LCLAT1 knockdown BT183 cells against ki67 (green) and TOMM20 (red). Images were acquired at 100x magnification using an inverted Evos M5000 fluorescence microscope. Scale bars = 10 µm. (G) Relative expression of respiratory complexes I-V in control and LCLAT1 knockdown BT183. (H) Western blot analysis of key glycolysis enzymes. (I) Western blot analysis of tumor suppressors p53 and p21. (J) Western blot analysis of Sox2 and N-Myc. (K) Western blot analysis of LIN28A expression in control and LCLAT1 knockdown BT183 lysates on d3 and d7 post initial transfection. (L) Western blot analysis of Dcx expression on d3 and d7 post initial transfection. Western blot data represent the mean fold change in protein expression in Negative Control relative to LCLAT1 knockdown BT183 cells ± SD (*n* = 4). *P < .05, **P < .01, ***P < .001, ****P < .0001.

To further elucidate the functional role of LCLAT1-driven CL acyl chain remodeling in ETMR, we performed siRNA-mediated LCLAT1 knockdown (KD) in BT183 cells. Cells were transfected multiple times and collected on d3, d7, or d10 post-initial transfection to determine the KD efficacy of the mitochondrial-endosomal transmembrane protein. Western blot confirmed efficient reduction of LCLAT1 levels for all transfection schedules and time-points (Figure 6A;  Supplementary Figure S12). To mimic the effects of steady-state drug exposure and to assess the phenotypic and molecular changes under sustained knockdown, all downstream analyses were conducted at d10 post-initial transfection.

We first assessed the effects of LCLAT1 knockdown on CL profiles using MALDI-MS. Our results show both a structure-dependent reduction of CL species and a complete loss of several CLs, following LCLAT1 KD (Figure 6B). A more detailed profile of the alterations in individual CL acyl chain species is shown in Supplementary Figure S13.

We then examined the impact of LCLAT1 depletion on cell growth and proliferation (Figure 6C and D). LCLAT1 KD significantly reduced ETMR spheroid growth, measured over a 5-day period, with >100 µm difference in spheroid size recorded at day 5 post-seeding (Figure 6C). In 2D monolayers, cell confluence and total cell numbers were significantly decreased following LCLAT1 KD. Ki67^+^ labeling index also showed a reduction, demonstrating reduced proliferative activity following LCLAT1 KD (Figure 6D).

Given the known roles of CL and LCLAT1 in the regulation of mitochondrial dynamics, we examined whether LCLAT1 knockdown altered mitochondrial fission and fusion. Drp1 and its phosphorylated forms remained unchanged following LCLAT1 KD (Figure 6E). The expression of the pro-fission protein MFF was slightly decreased following LCLAT1 KD. Analysis of the pro-fusion proteins detected a significantly increased expression of Mfn1, while Mfn2 and Opa1 decreased. Collectively, these data demonstrate that LCLAT1-driven CL remodeling impacts the expression of both fission and fusion proteins in ETMR. These results are in contrast to previously published data in studies from non-CNS metabolic disorders, which demonstrates LCLAT1 KD or inhibition reduced Drp1 levels, while subsequently increasing Opa1 leading to increased mitochondria fusion.^32^^,^^47^^,^^48^ To evaluate the impact of LCLAT1 KD and altered fission/fusion protein expression on mitochondrial morphology, we carried out ICC staining against TOMM20. Image analysis revealed increased mitochondrial elongation and networking, particularly among Ki67-low and Ki67-negative populations (Figure 6F).

We next assessed the effects of LCLAT1 knockdown on the expression of respiratory complex subunits and key glycolytic enzymes (Figure 6G and H, respectively). LCLAT1 knockdown induced a marginal increase in Complex II and a reduction in Complex IV expression, while no significant changes were observed in the expression of Complexes I, III, and V. Unexpectedly, the glycolytic enzymes HKI, HKII, and PFKP levels were markedly higher, while GAPDH and PKM2 levels were slightly decreased following LCLAT1 KD (Figure 6H).

Finally, as mitochondrial dynamics and bioenergetics are known to regulate stem cell self-renewal and differentiation, we sought to investigate the impact of LCLAT1-driven CL remodeling on oncogenic, tumor suppressor, and neuronal differentiation pathways (Figure 6I–L). Following LCLAT1 KD, we detected increased levels of the tumor suppressors p53 and p21 (Figure 6I). Consistent with this, we detected moderate but significant reductions in N-Myc and Sox2 (Figure 6J). Interestingly, despite the overall anti-oncogenic profile, LIN28A expression was robustly increased, with a 2.1-fold increase detected at d3 and an 11.7-fold increase by d7 (Figure 6K).The increase in LIN28A correlated with an increase in the early neuronal differentiation marker, doublecortin (Dcx). Dcx expression was elevated in a temporal pattern paralleling LIN28A induction, showing a 1.5-fold increase on d3 and a 2.6-fold increase by d7 (Figure 6L).

### Targeting Drp1 and Mitochondrial Fission in ETMR

To further demonstrate the therapeutic potential of modulating the mitochondrial phenotype detected, we targeted the mitochondrial fission protein Drp1, which we showed to be upregulated in ETMR. Treatment of BT183 spheroids with the Drp1 inhibitor Mdivi-1^49^ was effective at reducing embryonal tumor cell viability (IC_50_ = 20.64 µM) in a dose-dependent manner (Supplementary Figure S14A). These results are in agreement with previously published preclinical efficacy data in glioblastoma^50–52^ and neuroblastoma.^53^^,^^54^ Conversely, treatment of NSC spheroids using the same Mdivi-1 dose range as in BT183 resulted in incomplete inhibition of viability, demonstrating a more tumor stem cell-specific therapy (Supplementary Figure S14B).

## Discussion

Clinically, ETMR is among the most difficult pediatric tumors to treat.^4^ Retrospective analyses have identified a combination of radiation therapy, gross total resection, and high doses of chemotherapy to be the most effective treatment for ETMR.^4^^,^^6^^,^^7^ While this combination has demonstrated an increase in curative outcomes in this aggressive tumor, its effect on the developing brain should not be understated. Young children who survive ETMR face serious life-long sequelae from the toxic therapies used to combat their tumor. Therefore, there is a tremendous need for ETMR therapies that are both more effective and less harmful.

To identify potential new therapeutic targets based on tumor-specific dysregulated functional lipids, we conducted spatial analyses of patient samples, a patient-derived 3D ETMR model, and a control neural stem cell spheroid model. We found alterations in cardiolipin composition that specifically accumulated in fragmented mitochondria of rapidly proliferating ETMR cells in both the patient samples and the BT183 cell model. Importantly, the accumulation of these CL species in ETMR cells directly correlated with the significantly increased proliferative capacity of BT183 tumorspheres, as compared to the NSC model. Our results indicate that mitochondrial fragmentation in ETMR is driven by increased levels of pro-fission enzymes (Drp-1, pDrp1 (Ser616), and MFF) and reduced levels of fusion enzymes (Opa1). Furthermore, ETMR cells showed reduced expression of OxPhos complexes, and a more glycolytic phenotype compared to the NSCs with elongated and interconnected mitochondria. We identified a significantly increased expression of the CL acyl chain remodeling enzyme, LCLAT1, which we hypothesize can be targeted as a less toxic treatment for ETMR.

Very few studies exist that have identified an accumulation of structure-specific cardiolipins within preclinical and clinical adult and pediatric brain tumors.^33–35^ Analysis of CL acyl chain composition from five different syngeneic mouse brain tumor models encompassing astrocytoma, ependymoblastoma, and glioblastoma demonstrated tumor-specific structural alterations that correlated with ETC abnormalities.^33^ The authors showed that an increase in shorter acyl chain CLs in ependymoblastoma resulted in impaired ETC efficiency and hypothesized that this was the driving force for these tumors utilizing glucose as their prime energy source. Our findings correlate with this preclinical study and a recent proteomics study, which demonstrated that ETMR patient samples had a low abundance of respiratory complex proteins.^55^ The accumulation of structure-specific CLs in ETMR is also in agreement with results recently published by our group and Krieger *et al* in adult glioblastoma.^34^^,^^35^ Correlative imaging analysis in the latter study also linked structure-specific CL acyl chain accumulation with increased fragmented mitochondria in glioblastoma.

Overexpression of LCLAT1 and subsequent CL remodeling, as observed in our ETMR samples and preclinical model, have been proposed to reflect pathogenic changes that drive multiple metabolic and aging-related diseases, including obesity, type 2 diabetes, Parkinson’s disease, cardiomyopathies, and cancer.^30^^,^^31^^,^^47^^,^^56^^,^^57^ The mechanistic pathways involved in pathological CL remodeling by LCLAT1 have primarily been investigated in these metabolic diseases and non-CNS tumors. For example, upregulation of LCLAT1 was shown to drive mitochondrial fission through the induction of Drp1, which led to subsequent impairment of mitochondrial respiration in a Parkinson’s disease model, similar to the results observed here for ETMR.^30^ In models of obesity, fatty liver disease, and myocardial infarction, upregulated LCLAT1 expression and subsequent CL remodeling resulted in the downregulation of ETC complexes, increased ROS production, activation of hypoxia-responsive signaling pathways, aberrant crista formation, and mitochondrial dysfunction.^48^^,^^56^^,^^58^ Ablation or pharmacological inhibition of LCLAT1 prevented mitochondrial dysfunction by restoring non-pathological CL profiles, improving mitochondrial ETC activity, suppressing Drp1 activation, and preventing the onset of obesity, type2 diabetes, myocardial infarction, fatty liver disease, and neurological diseases, as reviewed in Ref.^32^

In cancer studies, overexpression of LCLAT1 was recently identified as a prognostic marker for recurrence and survival rates in head and neck squamous cell carcinoma (HNSCC).^31^ The same group also reported increased LCLAT1 expression in CNS, gastric, lung, and prostate cancers using the ONCOMINE database. Similarly, LCLAT1 expression was recently found to be upregulated in hepatocellular carcinoma (HCC), compared to normal liver tissue, and along with AGPAT5 and LPCAT1, was identified as a highly sensitive biomarker that correlated with worse prognosis and overall survival.^59^ Knockdown of LCLAT1 in HCC cell models impaired tumor cell proliferation, migration, and invasion. This group also compared LCLAT1 gene expression in pan-cancer tissues compared to normal tissue using the XENA-TCGA-GTEx datasets, finding overexpression in many tumors, including glioblastoma. Increased expression of LCLAT1 and CL levels were also reported in patient samples and cell models of non-small cell lung cancer (NSCLC).^44^ This study demonstrated that LCLAT1 regulated cardiolipin composition and mitochondrial dynamics, promoting mitochondrial fission and reducing fusion. Knockdown of LCLAT1 induced changes in CL structures, increased mitochondrial fusion, and significantly reduced migration and proliferation of NSCLC cells in vitro and in vivo.^44^

We carried out siRNA KD of LCLAT1 to evaluate the functional significance of CL remodeling in ETMR biology. Following KD of LCLAT1, we show altered CL acyl chain composition that resulted in a significant reduction in cell growth and proliferation, in agreement with the findings in non-CNS tumors.^44^^,^^59^ LCLAT1 KD altered the expression profiles of proteins that regulate mitochondrial dynamics and partially restored mitochondrial elongation and networking, which was more pronounced in Ki67- low and negative cells. Interestingly, this appeared to occur independently of Drp1 expression or phosphorylation status, which is in contrast to previously reported studies that show that LCLAT1 inhibition regulates Drp1 recruitment and activation.^30^^,^^32^ Instead, it was associated with increased Mfn1 expression in the context of reduced Mfn2 and Opa1. Mitochondrial dynamics and bioenergetics have been intricately linked to the regulation of stemness and differentiation in both healthy and cancer stem cells.^45^^,^^46^^,^^51^ Although there are only a few studies specifically reporting LCLAT1/CL-driven mitochondrial dysregulation in cancer, mitochondrial fragmentation due to altered expression of fission and fusion proteins, coupled with enhanced glycolysis and chemoresistance, has been reported in numerous human cancers.^45^^,^^51^ This phenotype has been posited as a therapeutic target that can induce chemosensitivity, as reviewed in Refs.^45^^,^^60^ In medulloblastoma, sonic hedgehog signaling was shown to induce mitochondrial fragmentation through induction of Drp1 and suppression of the pro-fusion proteins, MFN1/2, in vitro and in vivo.^61^ The fragmented mitochondria in medulloblastoma also displayed aberrant cristae structure, similar to our findings in ETMR. In neuroblastoma cell models, the antiapoptotic protein, Survivin, was shown to recruit Drp1 to the mitochondria, resulting in fragmentation and metabolic reprogramming to aerobic glycolysis.^62^ Increased activity of the Survivin gene is associated with the gain of chromosome 17q in aggressive neuroblastoma tumors. More recently, mitochondrial fragmentation due to excess fission was shown to be mediated by enhanced Drp1 activation in brain tumor initiating cells derived from glioblastoma patients.^51^ We additionally demonstrate the potential of targeting the fragmented mitochondrial phenotype detected in ETMR by showing selective and dose-dependent reduction of cell viability and growth in BT183 tumorspheres following treatment with the Drp1 inhibitor, Mdivi-1.

While the expression of ETC subunits remained relatively unchanged following LCLAT1 KD, with slight increases in complex II and a reduction in complex IV, the impact on supercomplex formation, stability, and OXPHOS capacity were not measured at this time and warrant further investigation. Interestingly, of the glycolytic enzymes measured following LCLAT1 KD, significant increases were detected for HK I and II, and PFKP, whereas GAPDH demonstrated a significant reduction in expression. Increases in HKs and PFKP have been associated with increased growth, aggression, and proliferation in many tumors, including glioblastoma.^63^^,^^64^ We do not believe that to be the case here, as we detected a reduction in cell growth and proliferation. We also did not detect a concomitant upregulation in GAPDH, which has been suggested to be a pan-cancer marker that promotes growth, therapeutic resistance, and metastatic dissemination, as reviewed in Ref.^65^ A reduction in GAPDH with a concomitant decrease in ETMR cell growth correlates with previously published studies that show downregulation of GAPDH results in reduced tumor cell growth and proliferation.^66^^,^^67^

Further evidence for the anti-oncogenic effects of LCLAT1 KD in ETMR is shown by the increased expression of the tumor suppressors p53 and p21 in combination with the reduced expression of the oncoproteins, N-Myc and Sox2. The cyclin-dependent kinase inhibitor, p21, is a well-characterized transcriptional target of p53 that induces cell cycle arrest and apoptosis.^68–70^ The coordinated upregulation of p53^71^ and its downstream effector p21,^70^^,^^72^ along with a parallel decrease in ki67 LI, indicate that LCLAT1 depletion promotes partial cell cycle exit and arrest. Like p53, p21 also acts on a plethora of pathways and has been shown to reduce the expression of the pluripotency factor, Sox2, in a p53-independent manner.^73^ Sox2 is required for embryonic development, neurogenesis, and maintaining the pluripotency and stemness of embryonic and neuronal stem cells.^74^ Sox2 is highly expressed in many pediatric brain tumors, including ETMR,^55^ and its downregulation here, either directly by p21 or through other mechanisms, further supports the anti-oncogenic impact of CL remodeling by LCLAT1. The transcription factor N-Myc is required for normal brain development and is downregulated postnatally, with minimal expression in adult tissues.^75^ Its aberrant amplification is a well-established oncogenic driver in multiple pediatric tumors, including ETMR, where it has been identified as a distinct therapeutic vulnerability.^76^ Thus, the observed downregulation of N-Myc following LCLAT1 KD again supports the anti-oncogenic role of CL remodeling.

Interestingly, despite the overall anti-proliferative and anti-oncogenic signature observed upon LCLAT1 KD, expression levels of the pluripotency factor, LIN28A, were robustly upregulated. This was unexpected, as LIN28A overexpression is a diagnostic marker for ETMR and a known oncogenic driver tightly associated with stemness and proliferation, which is the opposite of what we observed following LCLAT1 KD. For instance, Sox2 was shown to regulate the expression of LIN28 in embryonic neural precursor cells,^77^ but in our studies, LCLAT1 KD reduced Sox2 expression, yet significantly increased LiIN28A expression. A similar dichotomy was observed for the known association of the C19MC-LIN28A-MYCN oncogenic circuit in ETMR,^76^ as again, N-Myc expression decreased, yet LIN28A significantly increased. It is also worth noting that overexpression of LIN28A neural progenitor cells in vivo is not sufficient to drive tumor formation,^78^ meaning LIN28A overexpression alone is not oncogenic. LIN28A is known to regulate metabolic processes, and many studies have highlighted its role as a glycolytic switch, demonstrating upregulation of HKII and down regulation of complex IV, which is in agreement with the expression profiles detected following LCLAT1 KD.^79^^,^^80^ LIN28A regulation of metabolism, however, is complex and appears to be dependent on the type of cell/tissue and environmental cues. For example, LIN28A is being studied in the field of regenerative medicine, and its overexpression has been shown to regulate both glycolytic and mitochondrial-based metabolic processes to drive tissue repair in multiple cells and organ systems.^81^^,^^82^ Somatic stem cells expressing LIN28A also demonstrated improved differentiation.^82^ While more in-depth functional studies are required to elucidate the mechanistic implications of LIN28A upregulation following LCLAT1 KD, the induction of LIN28A may reflect a shift in differentiation trajectory rather than a direct oncogenic response. This interpretation is further supported by our observation that LCLAT1 knockdown induced upregulation of double cortin, with temporal expression closely mirroring that of LIN28A. These findings go against the common narrative of LIN28A as a pluripotency factor but do agree with studies in neurogenesis and regenerative medicine, where LIN28A functions beyond pluripotency to facilitate neuronal fate transitions. For example, a previous study reported a 60% increase in the Dcx^+^ neuroblast population during differentiation of explanted NSCs following LIN28A overexpression.^83^ Developmental studies have shown that LIN28 is essential for brain development and cell fate succession by driving neuronal differentiation over glial differentiation and was thus postulated to not solely be a pluripotency factor.^84^ Studies in Parkinson’s disease have shown that LIN28A regulates midbrain dopaminergic (mDA) specific developmental genes for the production of healthy mDA neurons.^85^ Functional mutation in LIN28A resulted in a reduction in both mDA and GABAergic neurons, further highlighting potential roles of LIN28A in cell fate determination and function.^85^ Further studies to elucidate the functional outcome of LIN28A upregulation following LCLAT1 KD and CL remodeling are required, as we believe that the upregulation detected here is also due to roles outside of pluripotency.

Our study was limited by small sample numbers and only one primary model. We hope that in the near future there will be more ETMR models available as this is essential for advancing research to find curative treatments. Our data is possibly not specific to ETMR and could be a characteristic of aggressive pediatric brain tumors, this requires further study. Our results require validation in more ETMR models covering both in vitro and in vivo.

In conclusion, our findings provide new mechanistic insights into ETMR biology and its reliance on mitochondrial fission and enhanced glycolysis for proliferation, as well as establish CL remodeling via LCLAT1 as a regulator of tumor suppressors and oncogenic proteins. The parallel rise in LIN28A and Dcx is consistent with a shift toward neuronal differentiation, suggesting that modulating cardiolipin acyl chain composition could drive differentiation programs in ETMR. Given the known roles of mitochondrial dynamics and bioenergetics in driving cell differentiation, studies targeting CL remodeling and the mitochondrial phenotype detected in ETMR are an important next step of investigation.

## Supplementary Material

vdaf228_Supplementary_Data

## Data Availability

All original data from this manuscript will be made available upon reasonable request. MSI data will be uploaded to METASPACE once all data has been published.
