# Supplementary material for: LCLAT1 regulates cardiolipin composition, mitochondrial phenotype, Lin28A, and oncogenic signaling networks in ETMR
Source: Neurooncol Adv. 2025 Oct 21;7(1):vdaf228. doi: 10.1093/noajnl/vdaf228 (PMC12746605; doi:10.1093/noajnl/vdaf228)
Supplement: vdaf228_Supplementary_Data [file vdaf228_supplementary_data.zip › Supplementary Data_Clean.docx]

**Supplementary Data**

**
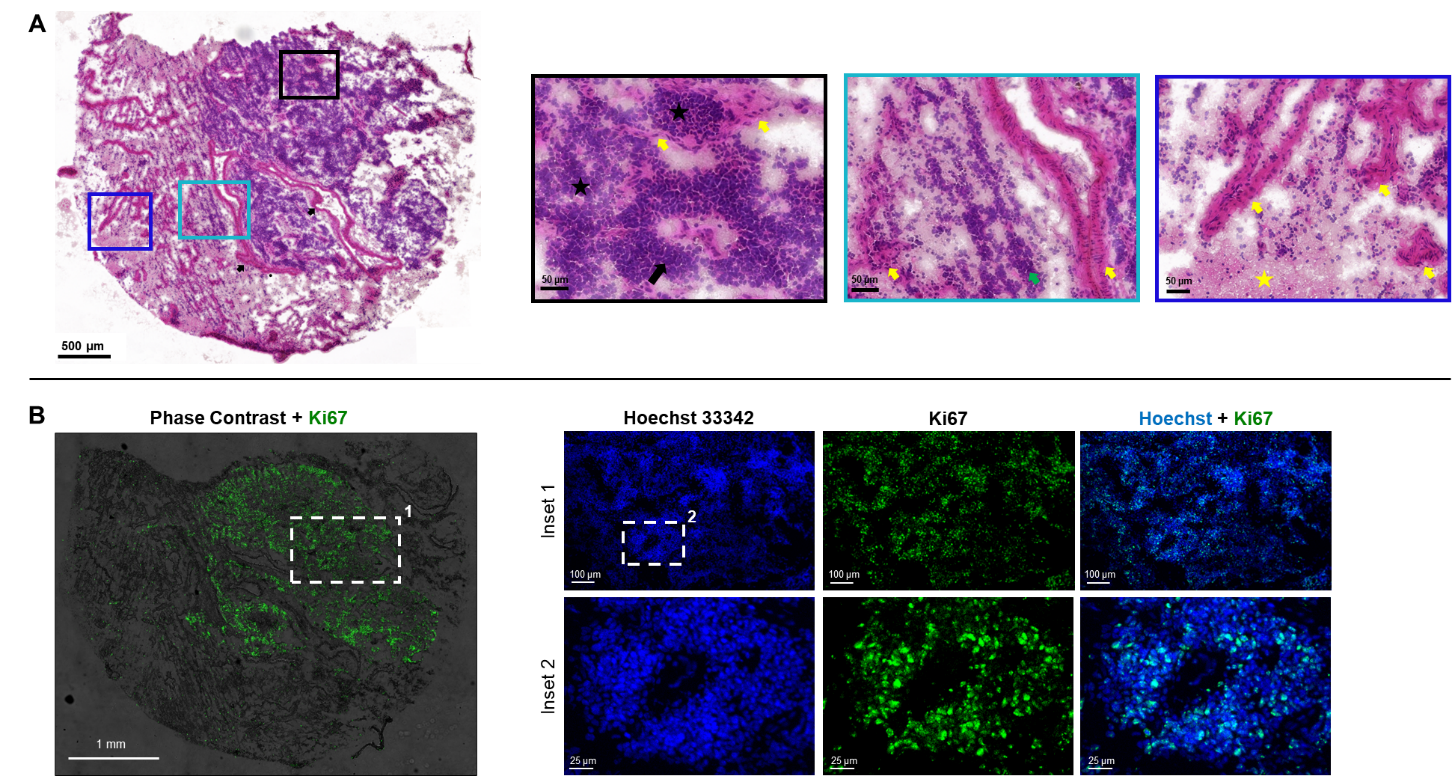
**

**Supplementary Figure S1**. **A)** H&E-stained section taken from ETMR sample 1. Higher magnification images show regions of high and low tumor cell density (black and yellow stars, respectively), multilayered rosettes (black arrow), and vascular formations (yellow arrows). **B)** Ki67 immunostaining in tumor cell populations; inset 2 shows Ki67 positivity in tumor cells forming multilayered rosettes.

**
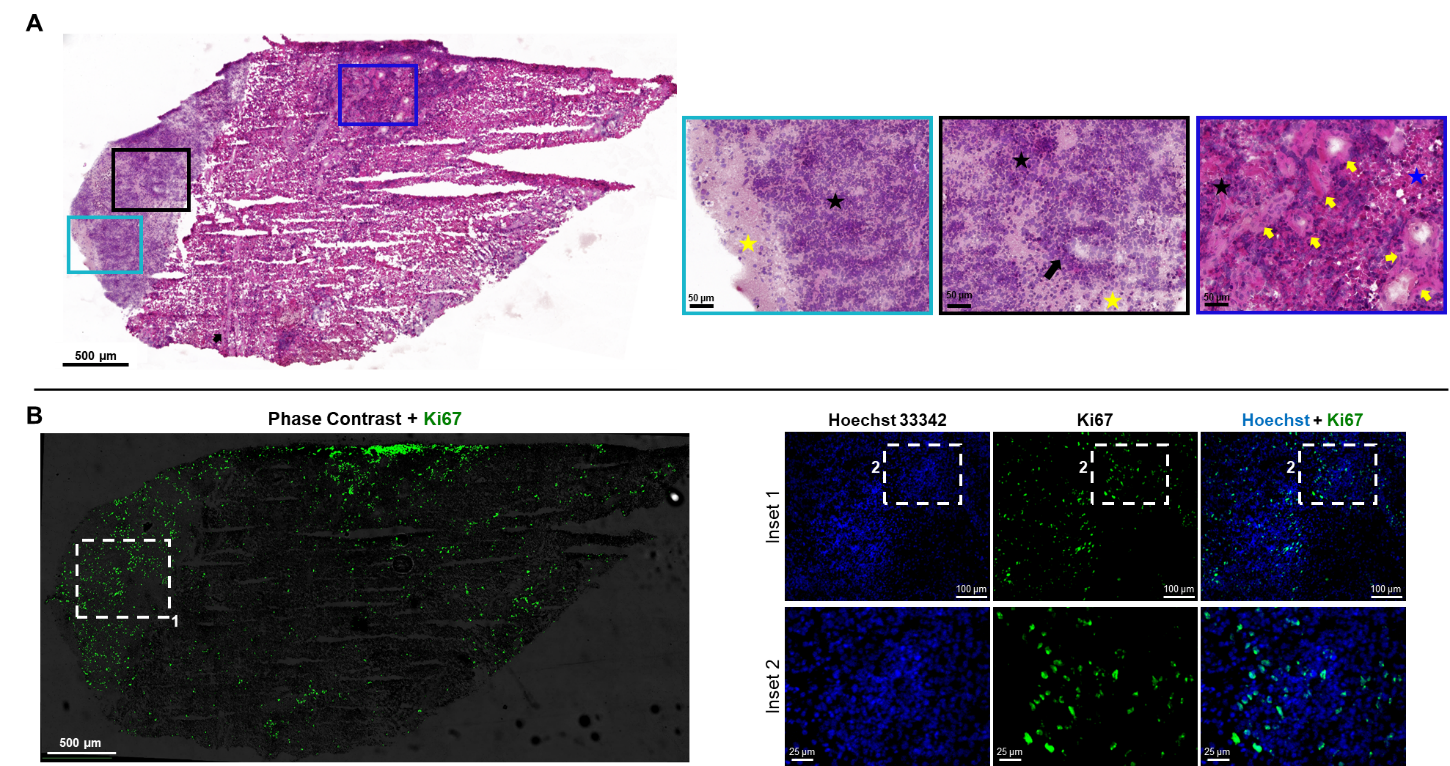

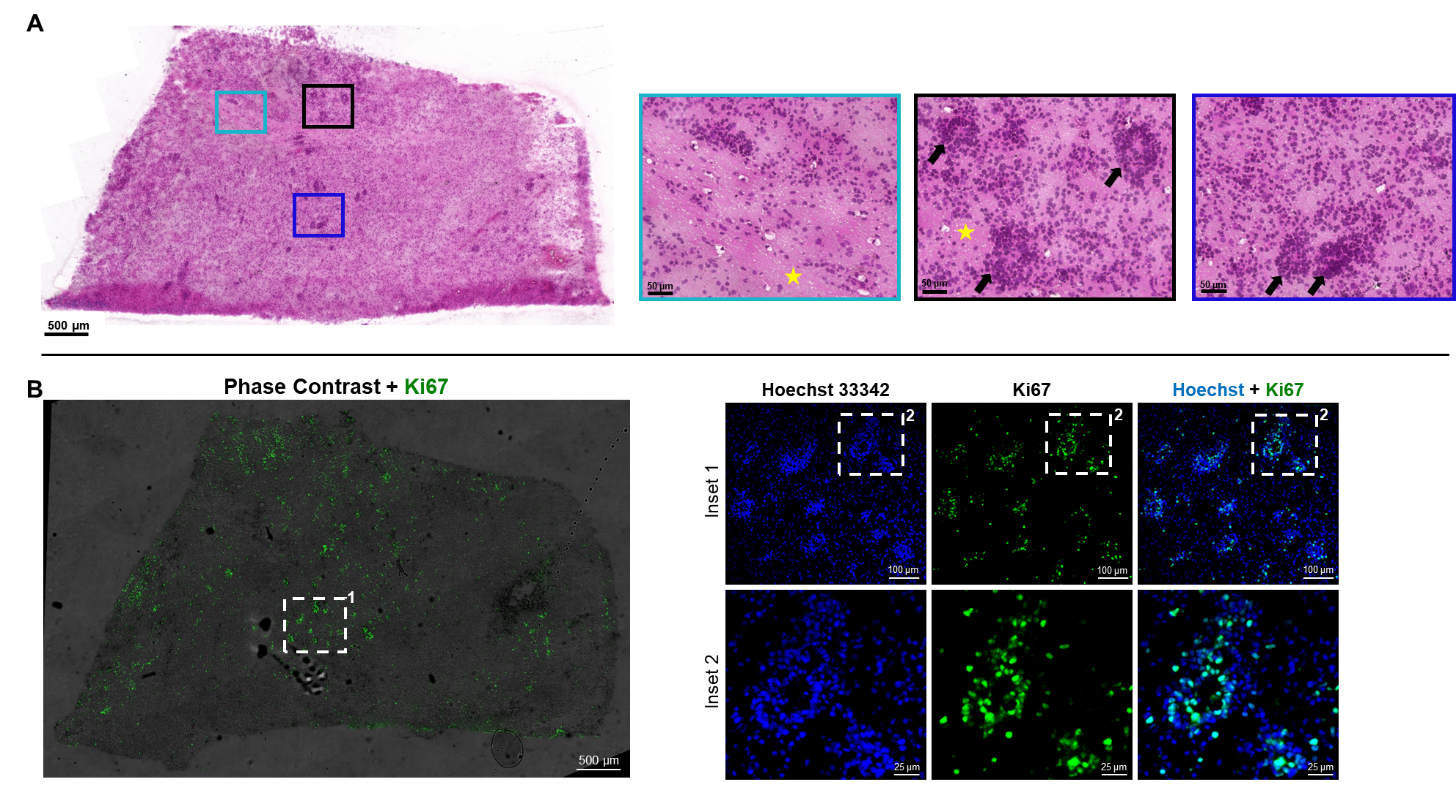
Supplementary Figure S2**. **A)** H&E-stained section taken from ETMR sample 2. Higher magnification images show regions of low tumor cell density with normal-appearing neuropil (yellow stars), multilayered rosettes (black arrows). Clusters of small blue embryonal tumor cells are present throughout the section. **B)** Ki67 immunostaining. Positive cells can be seen in the multilayered rosettes (inset 2) and in cells throughout the section.

**Supplementary Figure S3**. **A)** H&E-stained section taken from ETMR sample 3. Higher magnification images show regions of low tumor cell density with normal-appearing neuropil (yellow stars), multilayered rosettes (black arrows), and high-density regions of small blue embryonal tumor cells that are present throughout the section. Dense regions of vascular formations are present at the top of the section (blue box, yellow arrows), and a large region of necrosis is present throughout the section (blue star). **B)** Ki67 immunostaining shows strong positivity in the tumor cell regions to the left and top.

**
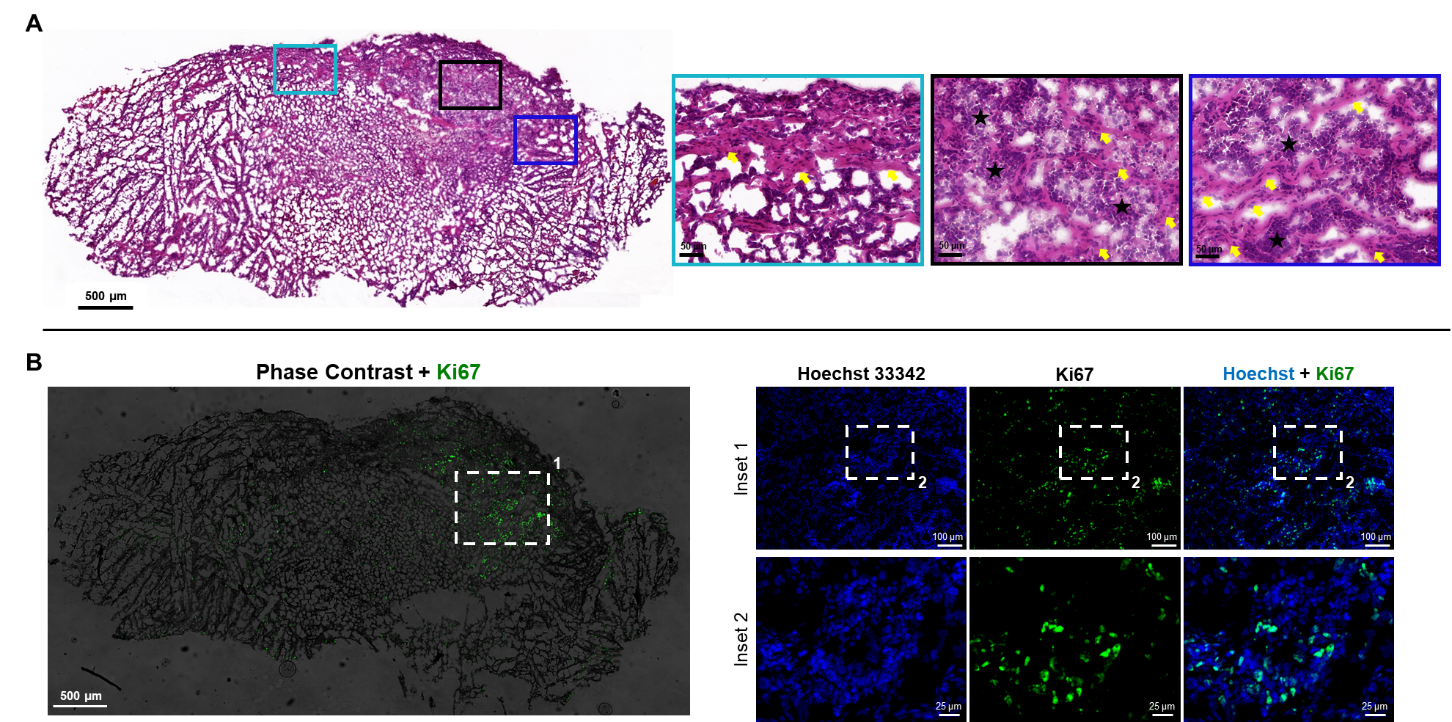
**

**Supplementary Figure S4**. **A)** H&E-stained section taken from ETMR sample 4. Higher magnification images show regions of abundant vascular formations (yellow arrows) surrounded by clusters of tumor cells (black stars). **B)** Ki67 immunostaining shows strong positivity within these cellular clusters. This section has freeze artifacts through the rest of the section.

**
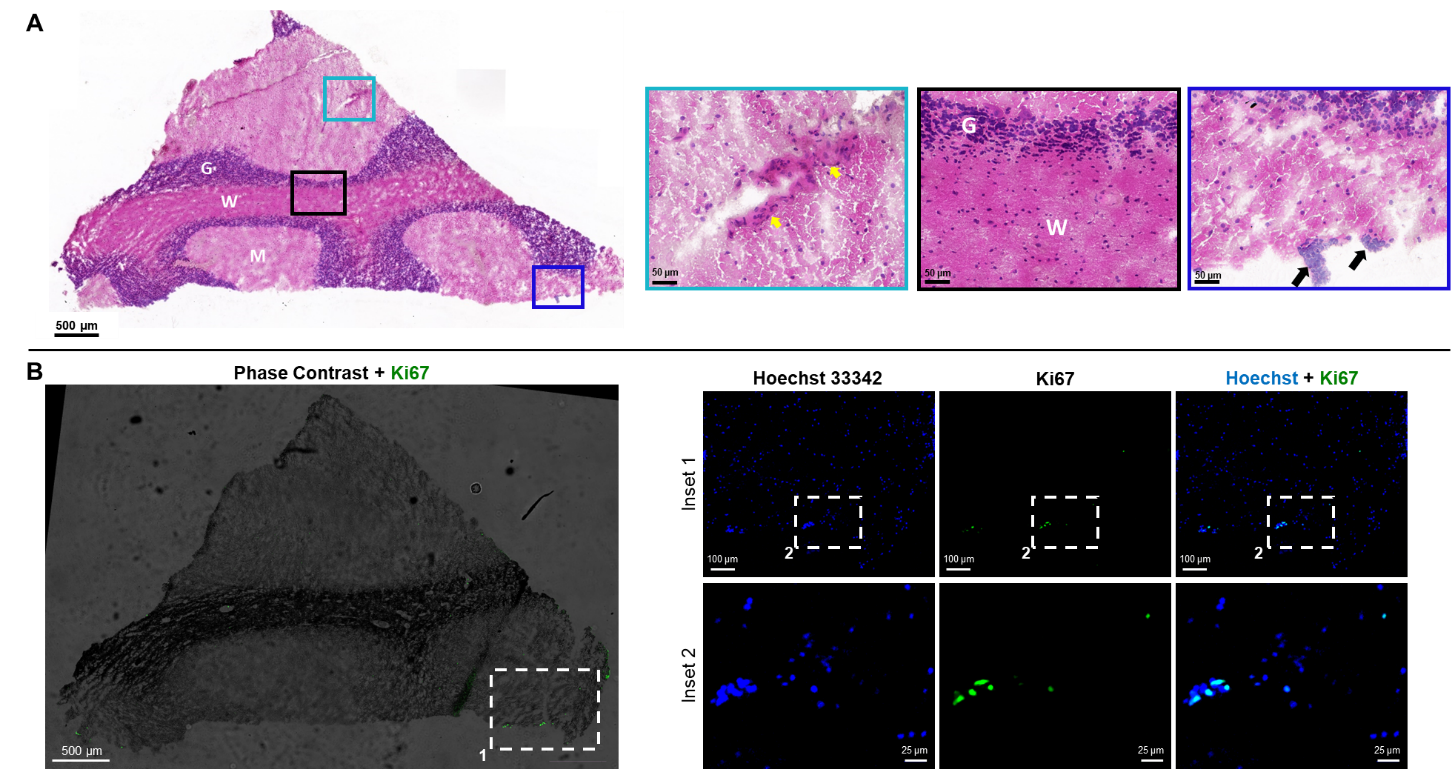
Supplementary Figure S5**. **A)** H&E-stained section taken from ETMR sample 6. Histology presents a mostly normal-appearing cerebellum; the molecular cell layer (M), granular cell layer (G), and white matter (W) are identifiable. Higher magnification images show vascular proliferations (yellow arrows) and a cluster of tumor cells in the bottom right (black arrows). **B)** Ki67 immunostaining shows a few positive cells in the bottom right of the section.


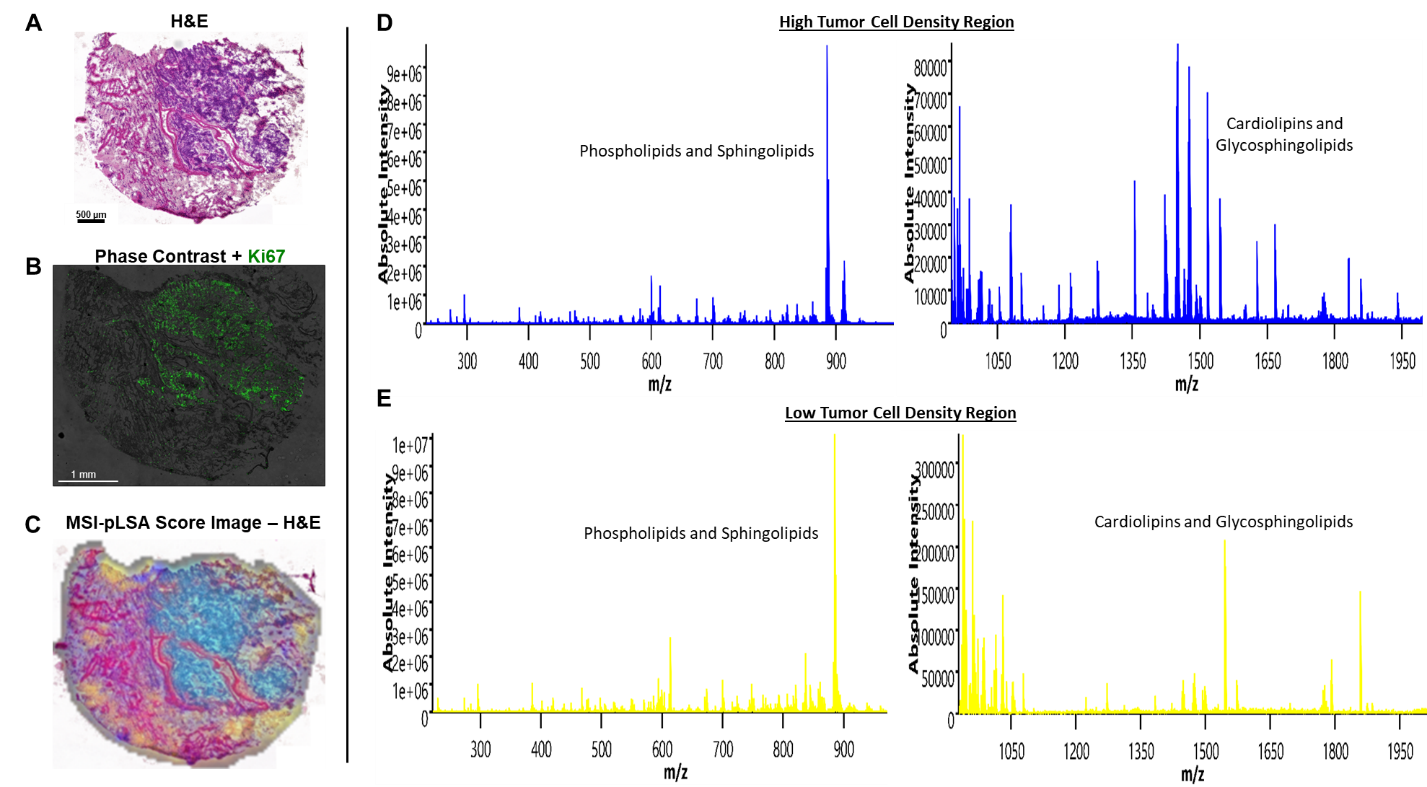


**Supplementary Figure S6.** Example data demonstrating molecular complexity within histologically defined regions and correlation to proliferation index in ETMR sample 1. **A)** H&E-stained section. **B)** Ki67 immunostaining. **C)** MALDI MSI merged score images from the pLSA analysis. Extracted mass spectra from the pLSA score images taken from **D)** high-density and highly proliferating regions (blue spectra) and **E)** low tumor cell density and low proliferation regions (yellow spectra).


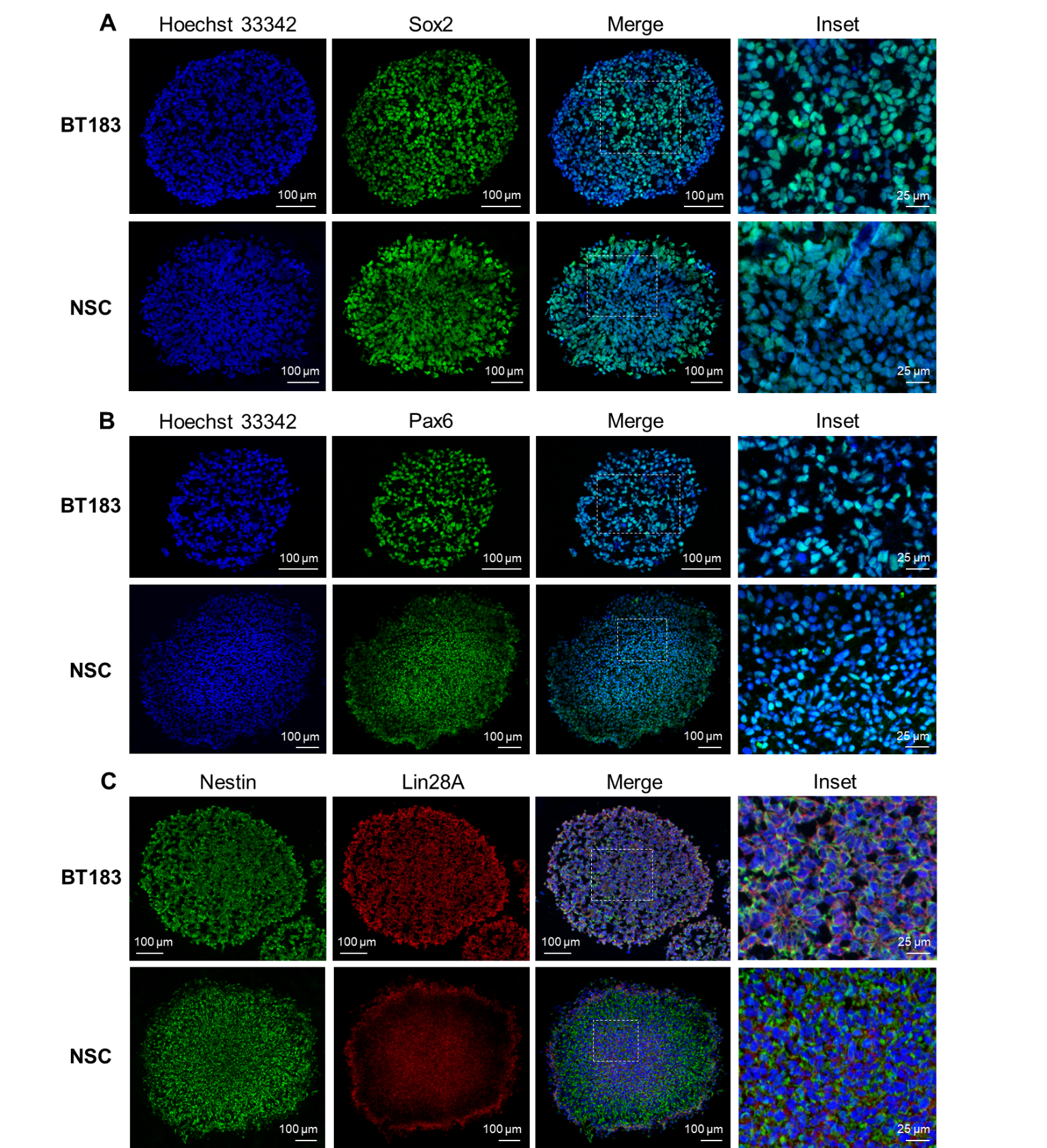
**Supplementary Figure S7.** Immunohistochemical characterization of NSC and BT183 cells grown as 3D neurospheres. IHC staining of BT183 and NSC neurosphere cryosections against **A)** Sox2 (green), **B)** Pax6 (green), **C)** Nestin (green) and Lin28A (red). BT183 cells exhibit specific immunoreactivity against these neural stem and progenitor markers, particularly at core tumorsphere regions and in “rosette”-like structures (insets). Hoechst 33342 was used as a nuclear counterstain. Images were acquired at 10x and 40x (insets) magnification using an inverted Evos M5000 fluorescence microscope. Scale bars = 100 µm. Inset scale bars = 25 µm.

**
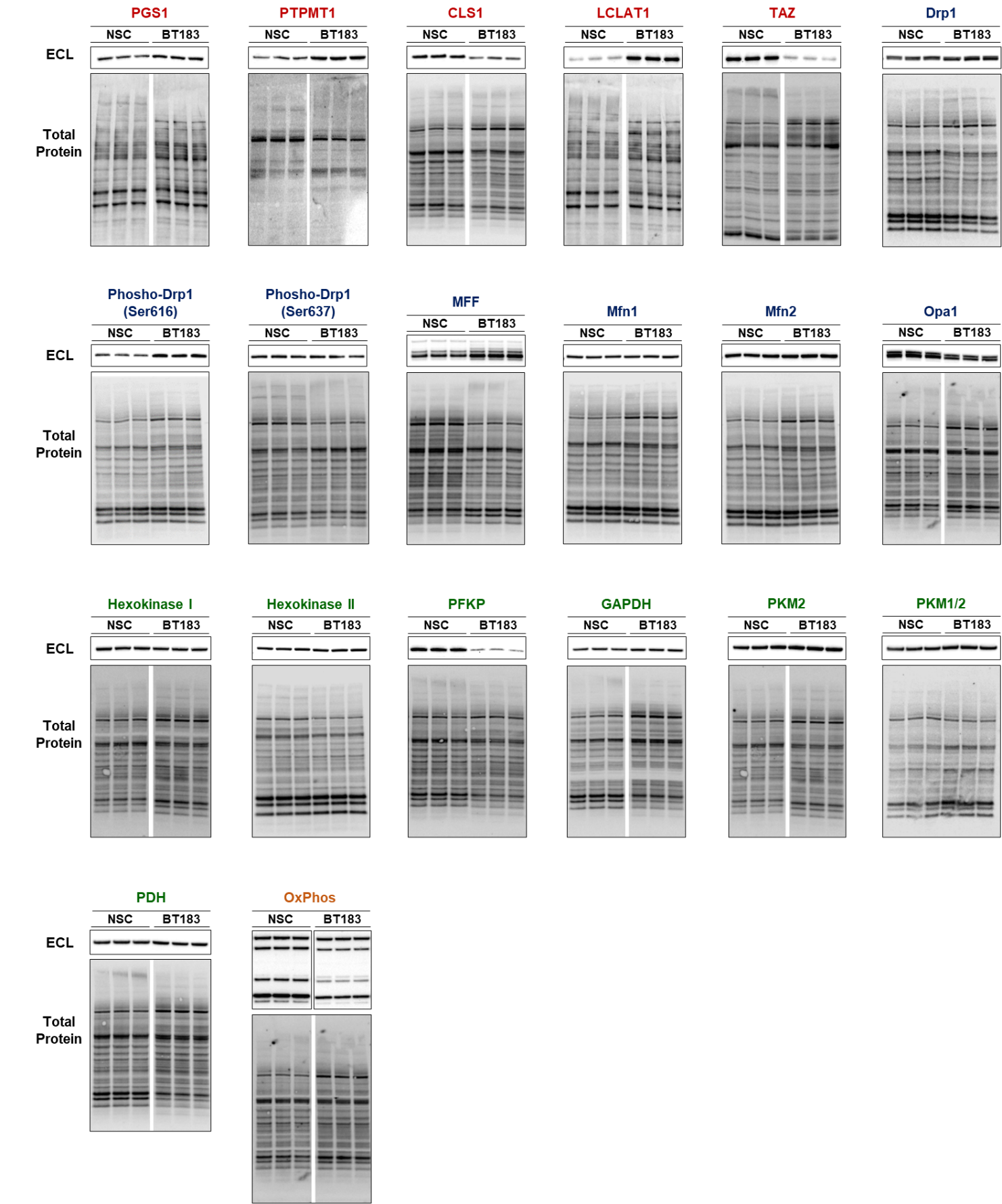
Supplementary Figure S8.** Enhanced Chemiluminescence (ECL) detection of protein markers described in the main text and total protein stains used as loading controls for normalization of BT183 vs NSC comparative western blot data.


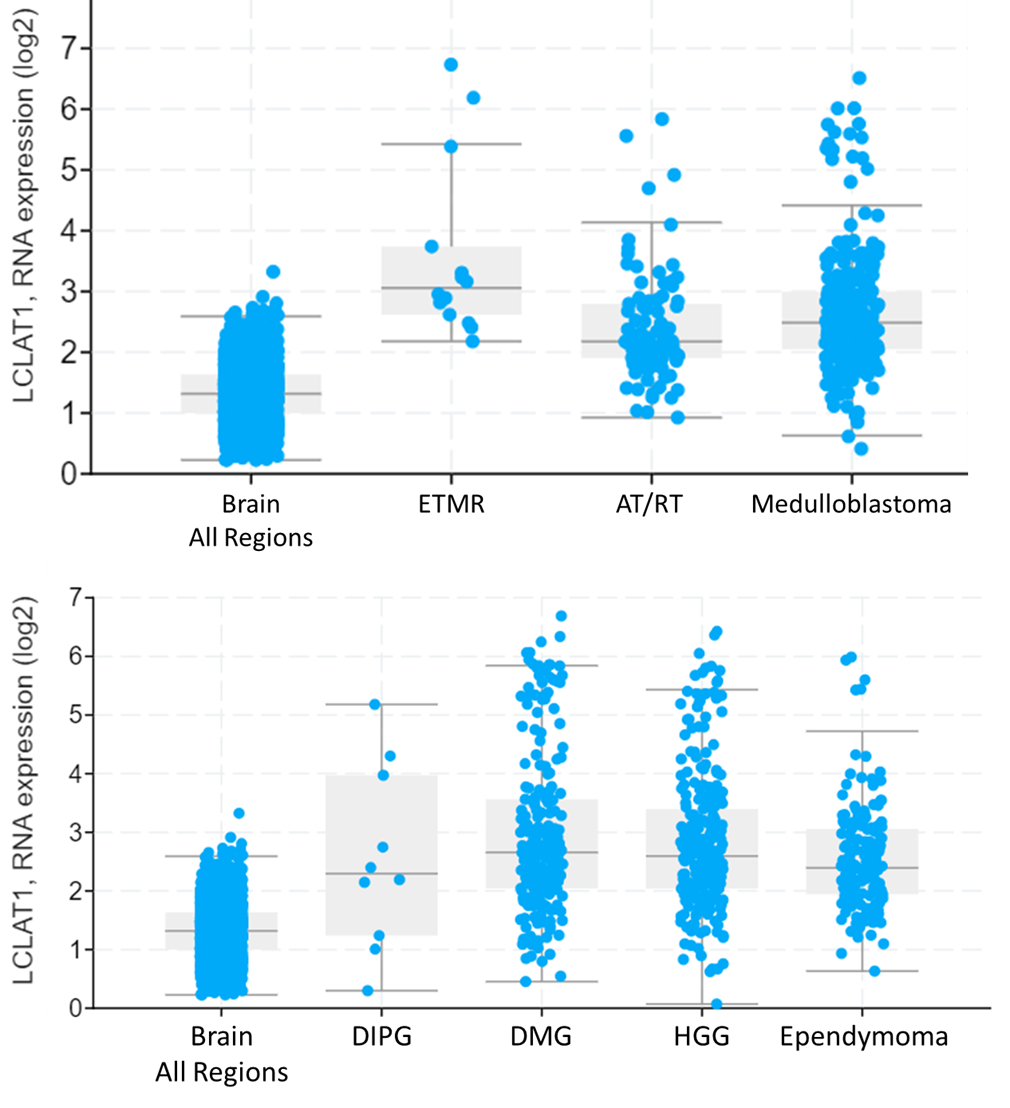


**Supplementary Figure S9.** LCLAT1 RNA expression data in normal brain vs. 7 pediatric brain tumors.


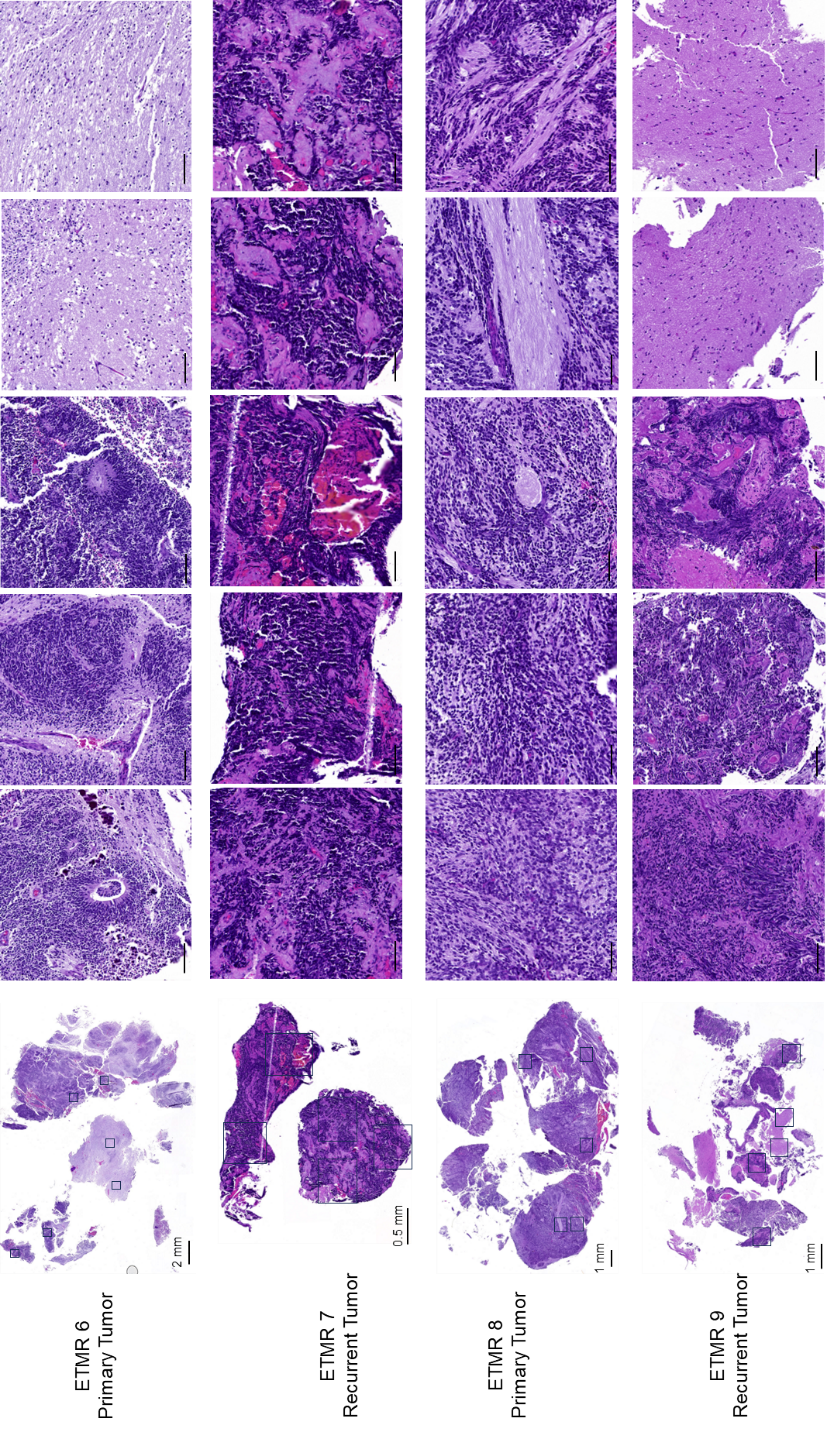


**Supplementary Figure S10.** H&E-stained sections of ETMR tumors 6-9, described in Figure 4 of the main text. Overview images were obtained at 40x magnification. Inset images were acquired at 15x zoom level. Inset scale bars = 100 µm.

**
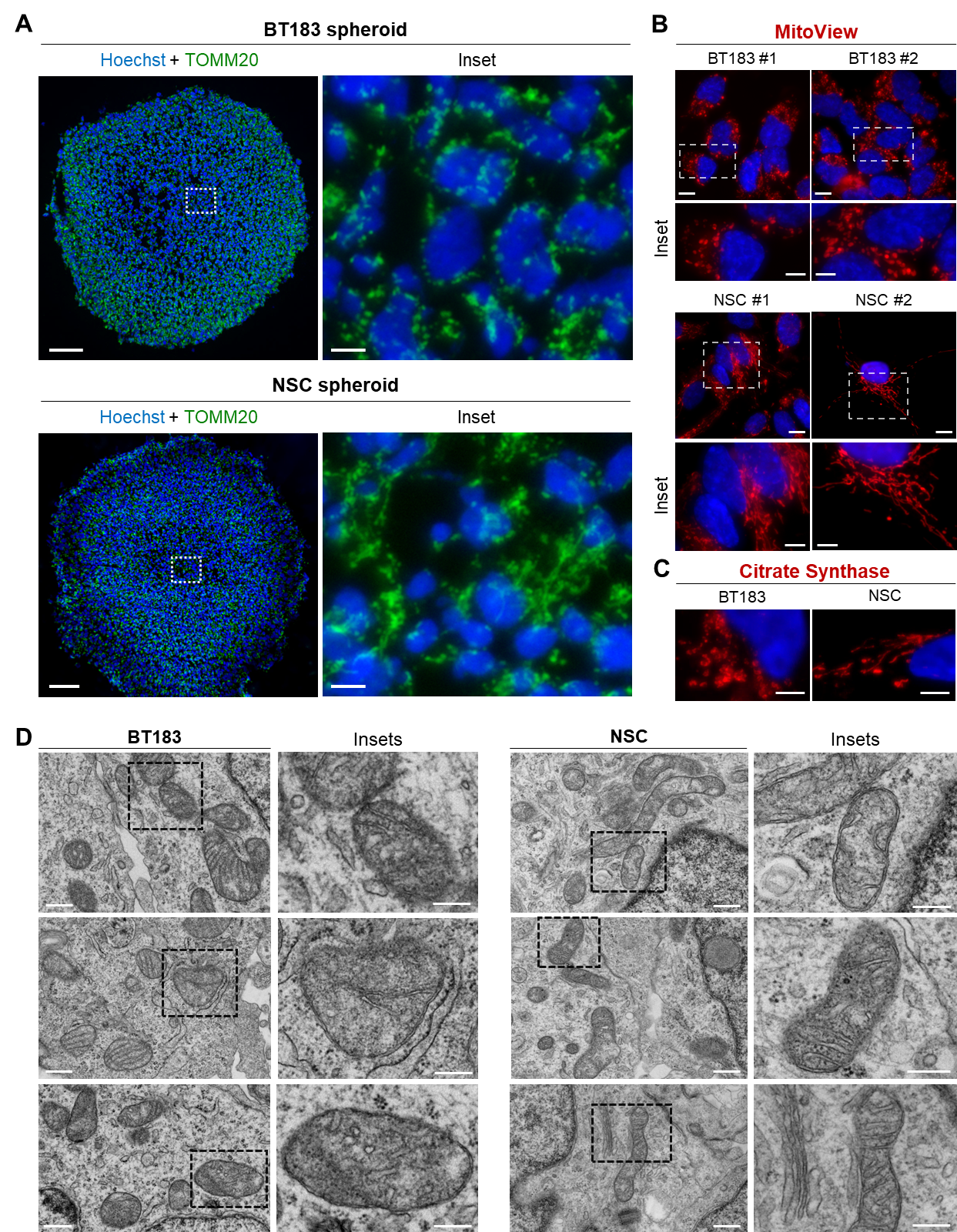
Supplementary Figure S11.** BT183 mitochondria display fragmented morphology. **A)** TOMM20-stained mitochondria in BT183 and NSC neurospheres. **B)** Representative images of mitochondria fluorescently stained with MitoView Fix 640 in BT183 and NSC cells grown as 2D monolayers in stem cell medium. Two representative fields of view for each cell type are presented. Images were acquired using a Nikon Ti2 Eclipse inverted fluorescence microscope at 100x magnification. Scale bars = 10 µm. Insets: scale bars = 5 µm. **C)** Citrate synthase staining of mitochondria in BT183 and NSC cells grown as monolayers. Scale bars = 5 µm. Hoechst 33342 was used to visualize the nuclei in panels A-C. Scale bars = 100 µm. Insets: scale bars = 5 µm. **D)** TEM images of mitochondria in BT183 and NSC cells grown as 3D neurospheres. Three representative fields of view for each cell type are shown. Scale bars = 500 nm. Insets: scale bars = 250 nm.

**
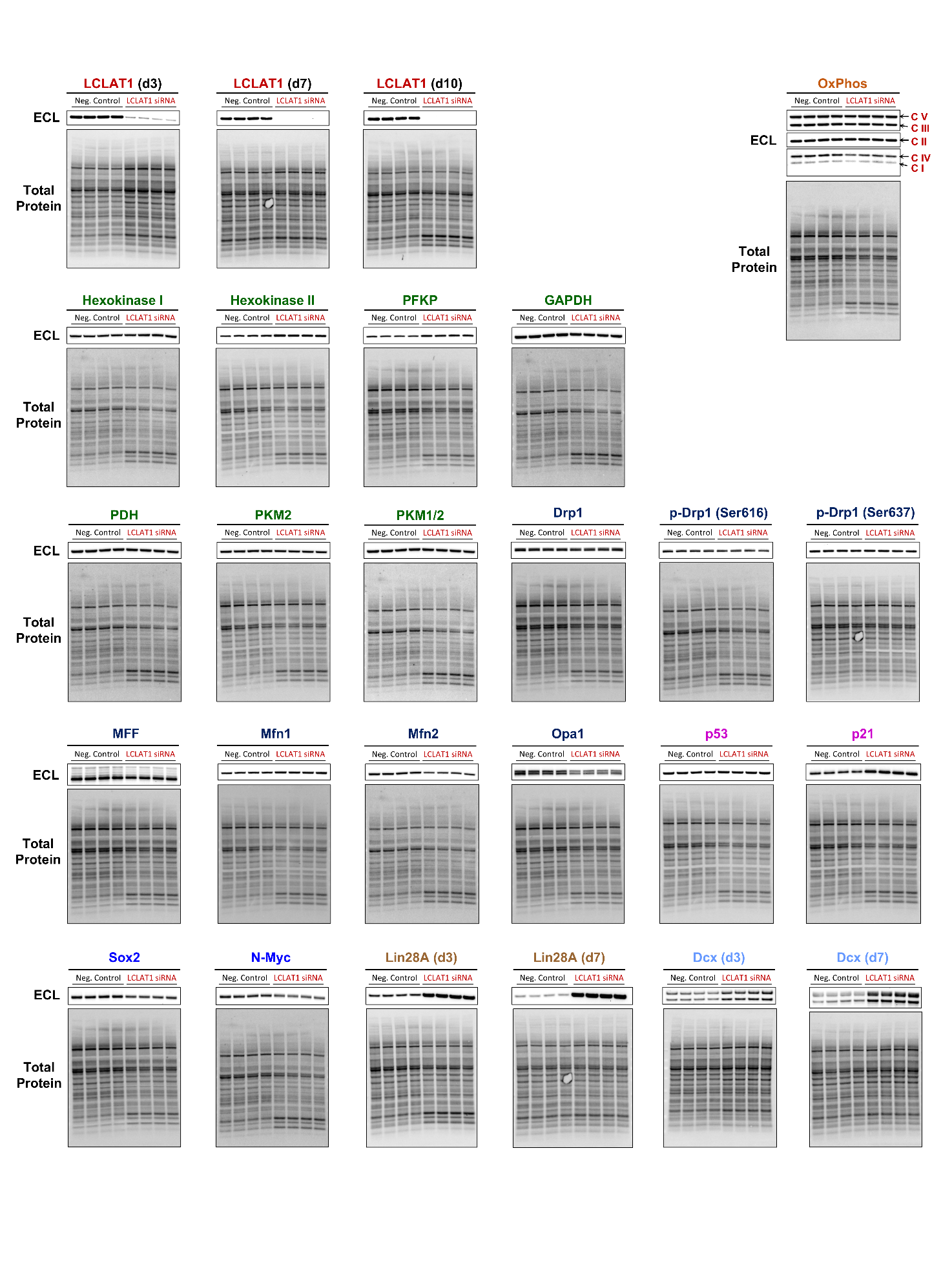
Supplementary Figure S12.** Enhanced Chemiluminescence (ECL) detection of protein markers and Total Protein stains used as loading controls for normalization of western blot data from LCLAT1 knockdown experiments.


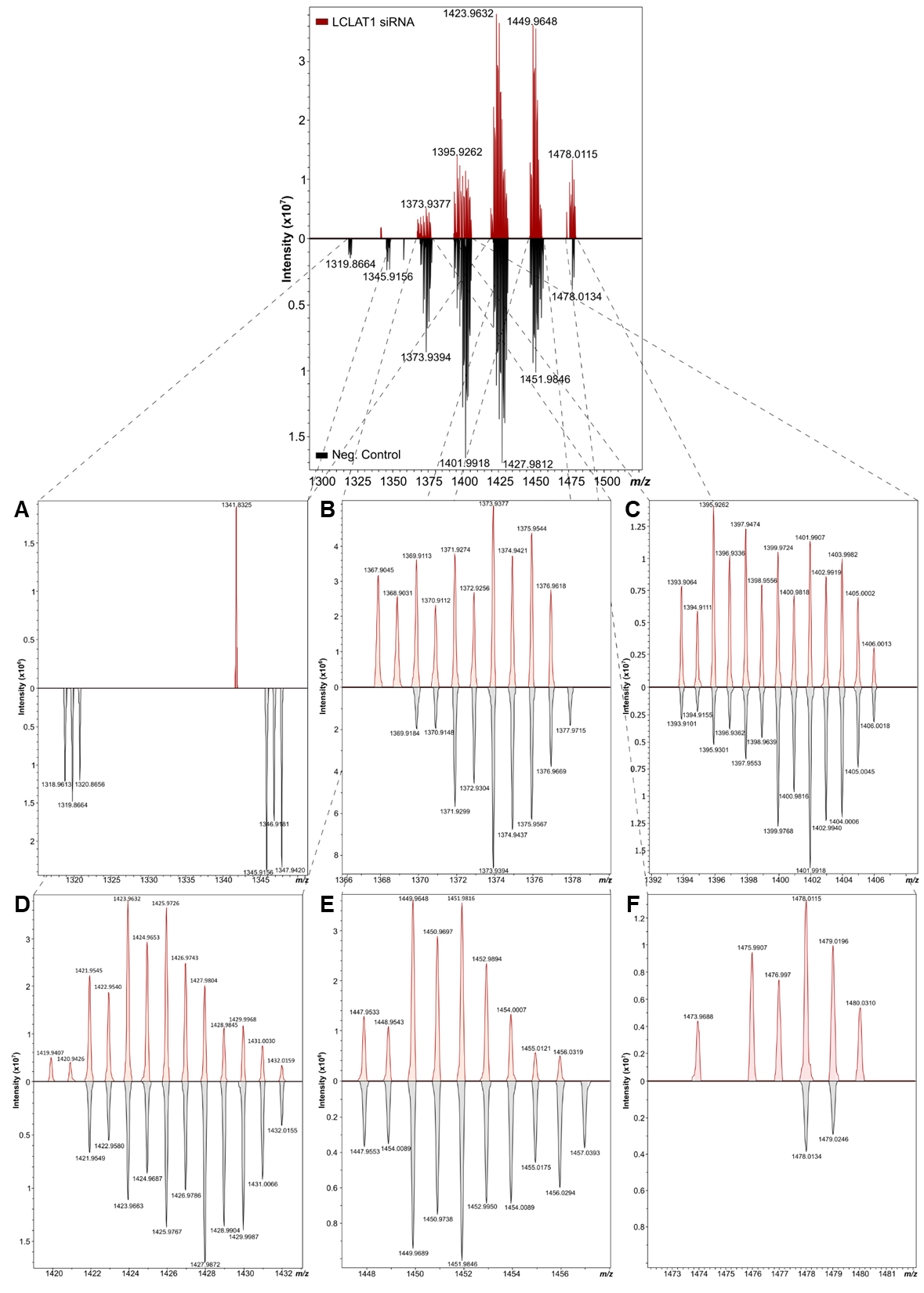
**Supplementary Figure S13.** Magnification of shorter **(A-C)** and longer **(D-F)** acyl chain cardiolipin profiles from LCLAT1 siRNA knockdown and negative control BT183 cells lipid extracts acquired by MALDI-MS.

**
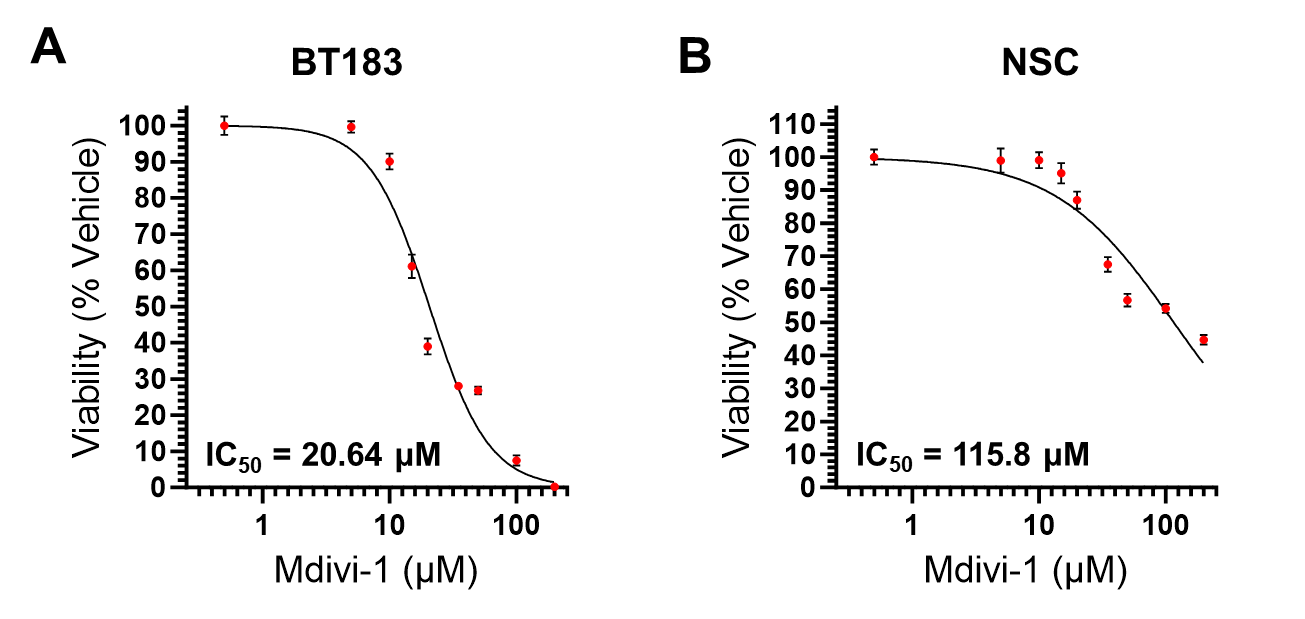
Supplementary Figure S14. A)** BT183 spheroid viability dose-response curve 72 h post-treatment with Mdivi-1, as determined by the Alamar Blue assay. Data points represent the mean spheroid viability from two independent experiments ± SEM (n = 6 spheroids per dose per plate). **B)** NSC spheroid viability dose-response curve 72 h post-treatment with Mdivi-1 determined by the Alamar Blue assay. Viability data are presented as mean ± SD (n = 6 spheroids per dose). IC50 values are displayed at the bottom left side of each graph. *p < 0.05, **p < 0.01, ***p < 0.001, ****p < 0.0001.
