# Supplementary material for: LCLAT1 regulates cardiolipin composition, mitochondrial phenotype, Lin28A, and oncogenic signaling networks in ETMR
Source: Neurooncol Adv. 2025 Oct 21;7(1):vdaf228. doi: 10.1093/noajnl/vdaf228 (PMC12746605; doi:10.1093/noajnl/vdaf228)
Supplement: vdaf228_Supplementary_Data [file vdaf228_supplementary_data.zip › Supplementary Materials and Methods_Clean.docx]

**Supplementary Materials and Methods**

**Patient Samples**

Frozen ETMR patient samples were serially sectioned at 10 µm thickness using a Leica 3050S cryostat and transferred to positively charged glass slides for histology, immunohistochemistry (IHC), and MSI. FFPE blocks were sectioned at 5 µm thickness using a Leica HistoCore microtome and transferred to positively charged glass slides for IHC.

**Cell lines and cell culture**

The ETMR cell line, BT183, was generously provided by Dr. Giselle L. Saulnier-Sholler (Penn State Health Children’s Hospital and Penn State College of Medicine). The NSC line was developed in-house by neural induction of a human pluripotent stem cell (PSC) line (iXCells Biotechnologies, San Diego, CA, USA) using Gibco™ PSC Neural Induction Medium (ThermoFisher Scientific, Waltham, MA, USA).

BT183 and NSC cells were grown in ultra-low attachment (ULA) flasks (Corning^TM^, Corning, NY, USA) in Complete NeuroCult medium, consisting of NS-A proliferation medium (STEMCELL Technologies Inc., British Columbia, Canada) supplemented with 20 ng/mL human epidermal growth factor (EGF) (ThermoFisher), 20 ng/mL heat stable human basic fibroblast growth factor (bFGF) (ThermoFisher), and 0.36 U/mL heparin (Sigma-Aldrich, St. Louis, MO, USA). All cells were cultured in a humidified incubator set at 37°C, 5% CO_2_.

***Ki67 labeling index scoring***

The Ki67^+^ labelling index (LI) in frozen tumor sections was determined using automated thresholding (Otsu method) and the “Analyze Particles” function in ImageJ (imageJ.nih.gov). Data were scored by two different methods: 1) the global method^1^, in which the percentage of Ki67^+^ tumor cells is estimated in whole tumor sections; and 2) the hot spot method^2^, in which the Ki67^+^ and Hoechst^+^ tumor cells are counted in tumor tissue areas displaying a particularly high density of Ki-67^+^ nuclei compared to adjacent regions. At least 12,000 cells per ETMR section for global analysis and 2,500 cells for hot spot analysis were used. Ki67 LI in BT183 and NSC neurospheres (n = 15) was estimated by automatically counting the percentage of Ki67^+^ cells in BT183 and NSC cryosections using the automated cell counting function in the Evos M5000 Imaging System software module (ThermoFisher).

**Preparation of BT183 and NSC spheroids for transmission electron microscopy (TEM)**

BT183 and NSC tumor/neurospheres were fixed in a solution consisting of 2.0% paraformaldehyde, 2.5% gluteraldehyde in 0.1M Cacodylate buffer. Spheres were fixed for 1 h at room temperature with gentle agitation. The fixative was replaced with 0.1M Cacodylate buffer, and samples were stored overnight at 4°C until ready for TEM processing and imaging.

**BT183 and NSC spheroid growth kinetics**

BT183 and NSCs were seeded in a round-bottom 96-well ULA plate (Corning) at a density of 5,000 cells per well. BT183 and NSC spheroids (n = 10 per cell type) were allowed to grow for up to 10 and 20 days, respectively. Phase contrast images of the 3D spheroids were taken at 2–3-day intervals using a Celigo Imaging Cytometer (Nexcelom Bioscience Waltham, MA, USA). Mean spheroid diameters were calculated from surface area measurements of spheroids performed in ImageJ (imageJ.nih.gov).

**Western Blotting**

Cells were lysed in RIPA Lysis and Extraction Buffer supplemented with 1x Halt™ Protease and Phosphatase Inhibitor Cocktail and 5 mM EDTA. Protein concentrations were measured using the Pierce™ Rapid Gold BCA Protein Assay Kit. Denaturing SDS-PAGE electrophoresis was performed using 4 to 12%, Bis-Tris, 1.0 mm, Mini Protein Gels using standard protocols. Proteins were then electroblotted on PVDF or nitrocellulose membranes using the iBlot™ 2 Gel Transfer or the Bio-Rad Turbo TransBlot. Membranes were incubated with primary antibodies overnight at 4°C and secondary antibodies for 1 h at room temperature. Chemiluminescent immunoreactivity and total protein content were detected using an iBright™ FL1000 or a Bio-Rad Chemidoc MP imager. Quantification of chemiluminescent signal intensity of individual protein bands and total protein normalization was performed using the iBright™ Analysis software. Relative protein expression is presented as mean fold change of protein expression in BT183 versus NSCs (control) ± SD (n ≥ 3 protein bands per cell type).

**Measurement of OCR and ECAR in BT183 and NSC spheroids**

The Oxygen Consumption Rate (OCR) and Extracellular Acidification Rate (ECAR) were determined using the MitoXpress® Xtra (MitoX) oxygen consumption and the pH-Xtra™ (pHX) glycolysis assay kits (Agilent), respectively, following the manufacturer’s recommendations. On the day of the metabolic assays, BT183 and NSC spheroids (n=6) were transferred to black (pHX assay) or transparent (MitoX assay) ULA 96-well plates (Corning). Time-resolved fluorescence (TRF) measurements of MitoXpress probe signal were performed using an M-Plex Infinite Pro 200 plate reader (Tecan), while changes in pH-Xtra signal were monitored by dual-read TRF using Spark plate reader (Tecan). Calculations of fluorescence intensity, lifetime slopes, and slope correction were performed using the Data Visualization tool (Agilent). Basal OCR and ECAR values for BT183 and NSC spheroids were normalized to spheroid diameter and are expressed as (RFU/h)/spheroid diameter (mm) and (µs/h)/spheroid diameter (mm), respectively.

**MitoView™ Fix 640 staining of mitochondria**

BT183 and NSC cells were grown overnight as monolayers in a 12-well plate on glass coverslips pre-coated with Geltrex (#A1413301, ThermoFisher) at a density of 50,000 cells/well. Live cells were stained with 100 nM MitoView™ Fix 640 (Biotium, Hayward, CA, United States) in culture medium for 5 h at 37°C, then fixed with 4% formaldehyde for 15 min at RT. The coverslips were briefly rinsed in PBS and mounted on glass slides using Prolong Glass Antifade Mountant (ThermoFisher).

**Microscopy and image analysis**

*IHC stained FFPE ETMR sections:* Images were acquired on a Leica Stellaris 5 confocal microscope (Leica, Wetzler, Germany) using Leica Application Suite X (LASX). Z-series spanning the entire ETMR section thickness (10 µm) were collected at a step size of 0.2 µm using a 63x HC oil immersion objective (NA = 1.4). 3D morphometric analysis of TOMM20-stained mitochondria was carried out using the LASX 3D Analysis software module. *IHC stained BT183/NSC spheroid sections:* Fluorescent micrographs were obtained at 10 - 40x magnification using an Evos M5000 inverted fluorescence microscope. *MitoView™ Fix 640 stained BT183 and NSC monolayers:* Fluorescent image z-stacks (n = 11 per cell type) were acquired at 100X magnification using a Nikon Eclipse Ti2 inverted fluorescence microscope. Automated quantitative 2D analysis of mitochondrial morphology and network characteristics was performed on a per-cell basis using the Mitochondria Analyzer plugin (https://github.com/AhsenChaudhry/Mitochondria-Analyzer) in ImageJ (imageJ.nih.gov).

**Spheroid growth kinetics in LCLAT1 knockdown cells**

Negative control and LCLAT1 knockdown cells were dissociated from their parent 6-well plates 7 days post initial transfection and re-seeded in a round-bottom 96-well ULA plate (Corning) at a density of 2,500 cells per well (n = 10 wells per sample). Spheroid growth was monitored over a period of 7 days. Phase contrast images of spheroids were acquired on day 2 post seeding and every 24 h onwards using Celigo Imaging Cytometer (Nexcelom Bioscience Waltham, MA, USA). Mean spheroid diameters were calculated from surface area measurements of spheroids performed in ImageJ (imageJ.nih.gov).

**Automated image analysis of cell confluence in LCLAT1 knockdown cells**

Cell confluence was determined by automated whole-plate phase contrast imaging using BioTek Cytation 10 plate imager (Agilent Technologies, Inc., Santa Clara, CA, USA) and subsequent analysis in BioTek Gen5 software module (Agilent). Images were acquired using a 4x objective and an 8 x 8 grid of fields of view in each well. Image tiles were stitched automatically to generate a single large image. Focusing was performed automatically using the integrated laser autofocus. The values for the parameters (e.g., thresholding, object size, etc.) used to set up the primary mask around the cells were determined empirically, following manufacturer’s recommendations and by visually confirming accurate placement of outlines around cells.

**Automated image analysis of total cell number and Ki67+ LI in LCLAT1 knockdown cells**

Negative Control and LCLAT1 knockdown BT183 cells were fixed in the 6-well plate on day 10 post initial transfection and stained against Ki67 for 3 h at room temperature, using standard immunocytochemistry procedures. Following, cells were incubated with 1 µg/ml Hoechst 33342 for 1 h at room temperature. The total number of Hoechst+ nuclei and percentage of ki67+ cells was estimated by whole plate automated imaging using BioTek Cytation 10 plate imager (Agilent Technologies, Inc., Santa Clara, CA, USA), equipped with DAPI, GFP and TRITC LED cubes and subsequent analysis in BioTek Gen5 software module (Agilent). Multichannel fluorescence images were captured at 4x magnification in widefield mode in an 8 x 8 image montage, encompassing the center of each well across the 6-well plate. Focus was achieved using the laser autofocus method. Image tiles were stitched automatically to create a single large image for downstream analysis and stitched images were background-subtracted before analysis. Optimal thresholding, size discrimination and object segmentation parameters were defined empirically per manufacturer’s recommendations.

***Mdivi-1 treatment of BT183 and NSC spheroids***

BT183 cells and NSCs were seeded in round bottom 96-well ULA plates (Corning) at a density of 2,500 and 5,000 cells per well, respectively. BT183 and NSC spheroids were treated with vehicle (DMSO) or Mdivi-1 (Sigma, #M0199) at a dose range of 5-200 μM when they reached a diameter of approx. 500 µm. Cell viability was measured after 72 h of treatment with Mdivi-1 using the Alamar Blue assay and a Tecan Spark microplate reader. BT183 and NSC viability dose-response curves and half-maximal inhibitory concentration (IC_50_) values were generated using a variable slope model in Prism (GraphPad Software, San Diego, CA). Data points in the BT183 sigmoid curve represent the mean spheroid viability from two independent experiments ± SEM (n = 6 spheroids per dose per plate), and in the NSC sigmoid curve the mean spheroid viability from a single experiment ± SD (n = 6 spheroids per dose).

Cell viability was measured after 72 h of treatment with Mdivi-1 using the Alamar Blue assay and a Tecan Spark microplate reader. BT183 and NSC viability dose-response curves and half-maximal inhibitory concentration (IC_50_) values were generated using a variable slope model in Prism (GraphPad Software, San Diego, CA). Data points in the BT183 sigmoid curve represent the mean spheroid viability from two independent experiments ± SEM (n = 6 spheroids per dose per plate) and in the NSC sigmoid curve the mean spheroid viability from a single experiment ± SD (n = 6 spheroids per dose).

**References**

**1.** Robertson S, Acs B, Lippert M, Hartman J. Prognostic potential of automated Ki67 evaluation in breast cancer: different hot spot definitions versus true global score. *Breast Cancer Res Treat.* 2020; 183(1):161-175.

**2.** Jang MH, Kim HJ, Chung YR, Lee Y, Park SY. A comparison of Ki-67 counting methods in luminal Breast Cancer: The Average Method vs. the Hot Spot Method. *PLoS One.* 2017; 12(2):e0172031.
